# Use of soluble guanylyl cyclase stimulators in heart failure therapy—a mode of action perspective

**DOI:** 10.1093/eschf/xvag117

**Published:** 2026-04-27

**Authors:** Frank Ruschitzka, Robert Lukowski, Stefano Corda, Christian Meier, Peter Sandner

**Affiliations:** Department of Cardiology, University Heart Center, University Hospital Zurich and University of Zurich, Zurich, Switzerland; Department of Cardiology, Center for Translational and Experimental Cardiology (CTEC), University Hospital Zurich, University of Zurich, Zurich, Switzerland; Institute of Pharmacy, Pharmacology, Toxicology and Clinical Pharmacy, University of Tübingen, Tübingen, Germany; BCC AG, Pharmaceuticals, Bayer Consumer Care AG, Basel, Switzerland; Global Medical Affairs, Bayer AG, Berlin, Germany; Research & Early Development, Bayer AG, Pharmaceuticals, Aprather Weg 18a, 42113 Wuppertal, Germany & Hannover Medical School, Hannover, Germany

**Keywords:** Soluble guanylyl cyclase, sGC stimulators, Vericiguat, Cyclic guanosine monophosphate, Heart failure

## Abstract

Despite recent advances in pharmacological treatment, chronic heart failure (HF) is associated with significant morbidity and mortality, and further treatment options are needed. Intact nitric oxide (NO)–soluble guanylyl cyclase (sGC)–cyclic guanosine monophosphate (cGMP) signalling is a prerequisite of cardiovascular health. cGMP produced by NO/NO–sGC acts as a second messenger molecule via various downstream targets, which influence a broad spectrum of critical physiological parameters. Impairment of this cascade in the cardiovascular system is considered an important pathomechanism in HF.

This review examines pharmacological therapies that act through the NO–sGC–cGMP signalling pathway. We will focus on the molecular mode(s) of action of NO-independent but haem-dependent sGC stimulators, and will examine evidence from preclinical studies demonstrating cardiovascular benefits of these therapies and their increasing number of effects on other susceptible tissues and organs, which together could contribute to clinical outcomes in HF.

The sGC stimulator vericiguat may be considered, in addition to standard therapy, for adults with symptomatic HF with reduced ejection fraction following a worsening event. The findings from pivotal clinical trials that led to these recommendations will be outlined and classified in terms of their significance for different subpopulations. These include the Phase 3 VICTORIA and VICTOR trials. Finally, further research areas and ongoing studies designed to address existing gaps in our knowledge regarding vericiguat and related drugs will be highlighted.

## Graphical Abstract

**Graphical Abstract:**
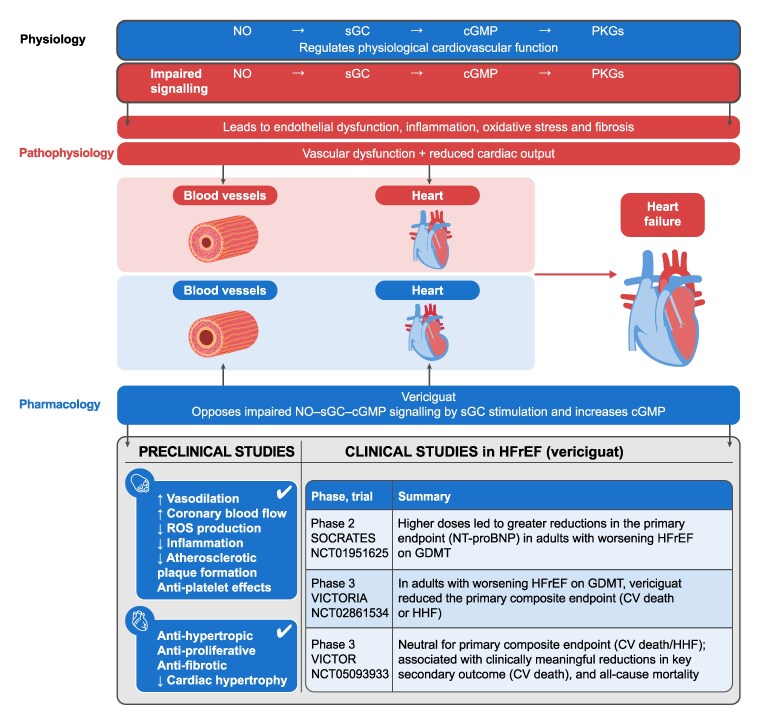
The role of sGC stimulation and of sGC stimulators in heart failure. GDMT at the time the study was performed. CV, cardiovascular; cGMP, cycle guanosine monophosphate; GDMT, guideline-directed medical therapy; HHF, hospitalization for heart failure; HFrEF, heart failure with reduced ejection fraction; NO, nitric oxide; NT-proBNP, N-terminal pro b-type natriuretic peptide; PKG, protein kinase G; ROS, reactive oxygen species; sGC, soluble guanylyl cyclase.

## Introduction

Chronic heart failure (HF) represents an important health care issue, with reports that prevalence may even be increasing in a steadily ageing population.^1–4^ Due to heterogeneous aetiologies and various comorbidities, treatment of HF remains challenging, and there is a substantial medical need for improved pharmacotherapy. Modulation of the renin–angiotensin–aldosterone system (RAAS) by angiotensin-converting enzyme inhibitors (ACEis), angiotensin-receptor blockers (ARBs), beta-blockers (BBs), mineralocorticoid receptor antagonists (MRAs), angiotensin-neprilysin-inhibitor (ARNi) therapy, and sodium-glucose cotransporter 2 inhibitors (SGLT2is), which are often used in certain combinations, improve outcomes in patients with HF with reduced ejection fraction (HFrEF).^5–7^ Recent progress in pharmacological treatment notwithstanding, HFrEF progression rates, morbidity and mortality remain high.^1^ Importantly, favourable effects of these drugs and their combination cannot easily be transferred to patients with HF with preserved ejection fraction (HFpEF), which is a condition triggered by obesity-induced metabolic stress as well as inflammation, hypertension, and ageing.^8^ This may be owing to the complex interplay of systemic and local signalling pathways and comorbidities, which result in different clinical HF phenotypes. It is interesting that for decades, HFpEF has proved to be a difficult entity to treat. Recent advances, particularly the introduction of SGLT2is, have demonstrated efficacy in reducing hospitalizations for HF and lowering cardiovascular (CV) mortality in this patient population.^9,10^ The precise disease-modifying mechanisms of action of SGLT2is in HF remain largely elusive. Thus, emerging treatment strategies with distinct mechanisms of action, when added to the current standard of care, hold the potential to enhance therapeutic efficacy even in patients already receiving foundational HFrEF or HFpEF therapies.

One molecular pathomechanism in chronic HF, driven by endothelial dysfunction, is an impaired nitric oxide (NO)–soluble guanylyl cyclase (sGC)–cyclic guanosine monophosphate (cGMP) signalling pathway.^11–14^ NO- and cGMP-based therapies have been known and investigated for decades. Early research demonstrated that NO, natriuretic peptides (NP) and cGMP all suppress both cardiac fibroblast production and cardiac myocyte hypertrophic growth.^15^ Following the initial publication over 30 years ago that described NO as the endothelial-derived relaxing factor and led to Robert F. Furchgott, Louis J. Ignarro, and Ferrid Murad being awarded the 1998 Nobel Prize in Medicine and Physiology,^16^ much research has been directed towards understanding NO and the intracellular signalling cascades mediated by cGMP. Recent evidence suggests that SGLT2is can interfere with the cardiac cGMP axis too.^17,18^ This emphasizes the urgent need to better understand the molecular and cellular effects of cGMP pathway modulation, especially in settings of steadily increasing concomitant medications. Presently available therapies, like organic nitrates or phosphodiesterase type 5 (PDE5) inhibitors, have limitations with no clear benefits on HF.^19–24^ Research over the last three decades led to the discovery of NO-independent sGC stimulators such as riociguat, which is beneficial in the treatment of pulmonary arterial hypertension (PAH; Group 1) and chronic thromboembolic pulmonary hypertension (Group 4), and eventually to the approval of vericiguat for the treatment of HFrEF.^25,26^ Since vericiguat is the first sGC stimulator approved in chronic HFrEF, this narrative review aims to discuss the NO–sGC–cGMP signalling pathway and the pharmacological therapies acting on this pathway, with a focus on vericiguat. For completeness and overview, other cGMP-enhancing drugs are covered, but with less detail. The molecular mode of action of sGC stimulation as a therapeutic target in HF is summarized in light of available clinical study results with vericiguat primarily in patients with HFrEF. This overview, based on authors’ expert knowledge, aims to direct future research across preclinical and clinical stages, with the objective of delineating how cGMP signalling interacts with the pathophysiological mechanisms of HF and its clinical sequelae.

## NO–sGC–cGMP signalling: a central player in regulation of cardiovascular homeostasis and heart function

As early as the 19^th^ century, physicians had demonstrated that application of amyl nitrate or glyceryl trinitrate leads to pronounced relief of symptoms in patients with angina pectoris. Although this was before the elucidation of the NO signalling pathway, it provided an early hint of how powerful the effect of enhanced NO-signalling can be for relaxing cardiac blood vessels. More than 150 years later, it is well established that intact NO–sGC signalling with sufficient cGMP supply is a major driver of CV health, and that impairment of this pathway leads to cardiopulmonary and cardiorenal dysfunctions.^11–13,27,28^ This knowledge comes from persistent and intense pharmacological preclinical profiling, and also from clinical investigations. Utilizing constantly developing genetic technologies, genome-wide association studies have not only confirmed, but substantially extended, the concept of impaired NO–sGC signalling as a driver of CV diseases.^29^ It was shown that genetic predisposition to impaired sGC function is associated with a higher risk of myocardial infarction (MI) and coronary artery disease (CAD).^30^ In contrast, genetically enhanced sGC activity reduces the risk of CAD, peripheral artery disease (PAD), or stroke.^31^ Mutations of sGC and other pathway components, such as NO-forming NO synthases (NOS) or cGMP-degrading phosphodiesterases (PDE), contribute to the overall CV disease phenotype.^29^

### Overview of the NO–sGC–cGMP cascade and related signalling components

Three isoforms of NO-synthases are known, which were traditionally named after their expression pattern: endothelial NO synthase (eNOS, NOS-3), neuronal NO synthase (nNOS, NOS-1), and inducible NO synthase (iNOS, NOS-2).^32^ Increased shear stress on blood vessel walls stimulates eNOS, inducing endothelial NO formation, whereas NO production in cardiomyocytes is primarily achieved by nNOS.^33^

**Figure 1:**
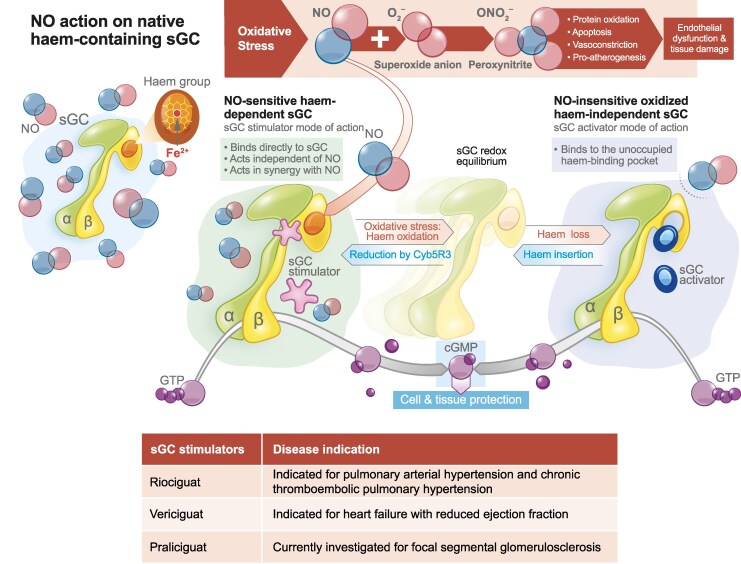
Schematic representation of the mechanisms by which sGC stimulators modify the NO–sGC–cGMP pathway.^26,35–37^ As illustrated, stimulators of sGC have two modes of action: (i) stimulate the native, haem-containing form of the enzyme, and (ii) increase the sensitivity of sGC to endogenous NO. The area on the right shows the action of sGC activators, which activate the oxidized, haem-free form of sGC. The actions of both sGC stimulators and sGC activators increase the formation of cGMP. α, alpha-sGC subunit; β, beta-sGC subunit; cGMP, cyclic guanosine monophosphate; Cyb5R3, cytochrome B5 reductase 3; Fe^2+^, haem ion; GTP, guanosine triphosphate; NO, nitric oxide; $O_{2}^{-}$, superoxide anion; ${\mathrm{ONO}}_{2}^{-}$, peroxynitrite; sGC, soluble guanylyl cyclase. Adapted from Sandner *et al.*^35^ under the terms of the CC-BY 4.0 license

Gaseous NO easily diffuses into adjacent vascular and non-vascular tissues and cells, such as smooth muscle cells and cardiomyocytes. Within the target cells, NO binds to the native, haem-containing sGC, leading to a conformational change and activation of the catalytic domain, triggering the formation of cGMP from GTP. The sGC haem iron is vulnerable to oxidation by oxidative stress, and cytochrome b5 reductase 3 (Cyb5R3) was identified to keep sGC in the reduced state in order to ensure NO-binding (*Figure 1*).^34^

The actions of NO in physiological vascular and cardiac function and performance are mainly attributable to its binding to the sGC enzyme and are cGMP-mediated.^13–16,35^ For more details on the CV NO signalling pathway, particularly the cGMP-independent effects mediated by NO, please refer to current reviews on these topics.^38–41^

cGMP stimulated by NO–sGC is a second messenger molecule with different downstream targets, including cGMP-dependent protein kinases (PKG; cGK). These mediate a broad range of effects by phosphorylating a large number of downstream targets, including but not limited to: calcium regulatory proteins, sarcomeric proteins, factors affecting gene expression, and even mitochondrial proteins.^40,42^ The wide and overlapping spectrum of sGC and PKG, as well as PKG target expression patterns, is likely a reason for the broad impact of NO on different cells and pathomechanisms in the CV system. The effects of this pathway extend beyond the ‘classical functions’ for vasodilation in different vascular beds. It is, for example, becoming increasingly clear that cGMP–PKG activity can also regulate cell motility, cell-to-cell interactions, cellular secretion, cell death and survival, as well as cell differentiation,^43^ which shows the great potential of pharmacological agents that interact with this cascade.

Termination of NO–sGC–cGMP signalling is exerted by cGMP-hydrolysing PDEs like PDE5 and 9. In addition to the different subcellular localization of the generators, the duration, amplitude, and distribution of a cGMP signal are governed by the cell’s PDE profile. Myocardial expression of PDE5 is low under normal physiological conditions, but up-regulated in HF.^40^  *In vivo* studies have demonstrated cardioprotective effects of PDE5 inhibition via effects on G-protein signalling, proteostasis, necrosis, and apoptosis, mitochondrial function, and sarcomeric proteins including titin.^40^ PDE5 inhibition down-regulates pro-hypertrophic and profibrotic microRNAs.^40^ Whether PDE5 exerts effects on cardiomyocytes remains unclear. PDE9 hydrolyses cGMP derived from the NP–pGC–cGMP pathway.^40^ PDE9 expression is up-regulated under certain disease conditions (e.g. HFpEF, aortic stenosis) while PDE9 inhibition has been shown to suppress cardiac hypertrophy in a rodent pressure-overload model.^40^ Emerging evidence points towards important functions of PDE1 and PDE2 in cardiac cells and tissues.^44,45^ However, the impact of PDEs, especially PDE5 and PDE9, is still highly controversial, and their cell type-specific functions will require special attention in the future. Furthermore, clinical trials with PDE5 inhibitors in HF, such as RELAX, failed to demonstrate clinical benefit in HFpEF.^22^

The NP–particulate guanylyl cyclase (pGC)–cGMP pathway is an alternative cGMP cascade for the regulation of CV and cardiorenal functions. pGCs are transmembrane receptors for extracellular peptides such as atrial, brain, or C-type NPs (ANP, BNP, and CNP, respectively).^12,46^ NPs regulate vascular smooth muscle tone, fluid and electrolyte balance, and have a protective role in the blood vessel wall.^47^ Despite acting through a common second messenger, cGMP, the NO–sGC and NP–pGC signalling pathways could also elicit different biological effects.^35^ This is made possible by the compartmentalization of the different cGMP pathways within the cell.^48,49^

However, the physiological functions of both cGMP-generating pathways also partially overlap, as stimulation of sGC and pGC can, among other effects, induce significant vasodilation. Moreover, both signalling pathways seem to play a role in pathological cardiac remodelling, which involves effects on structure, function, and energy metabolism of the heart induced by cardiac injury.^40,49–51^

Combination of sacubitril and valsartan administered as a single ARNi therapy (sacubitril-valsartan) was shown to be effective in the management of chronic HF, and has been approved for the treatment of HFrEF.^52,53^ The U.S. Food and Drug Administration expanded its indication for sacubitril-valsartan to chronic HF, stating the benefits are most clearly evident with an LV ejection fraction (LVEF) below normal (i.e. ≤40%).^53^ A detailed summary of NP–pGC–cGMP signalling and pharmacology is beyond the scope of this review; however, the reader is referred to the excellent overviews available on this topic.^51,54,55^

### Expression of the NO–sGC–cGMP pathway in the cardiovascular system

There is still some controversy regarding the role of NOS, sGC, PKG, and PDE expression in the CV system and in heart tissues. Quantitative expression profiles might also depend on the species and on disease states, and they may vary in patients with HF with a broad spectrum of comorbidities. However, cGMP is found in all major cardiac cell types: cardiomyocytes, cardiac fibroblasts, pericytes, vascular smooth muscle cells (VSMCs) and endothelial cells.^49,56^ sGC in cardiomyocytes regulates cardiac contractility and remodelling,^56^ while cardiomyocyte-specific NO–sGC signalling has demonstrated a crucial role in cardioprotection following acute MI in mice.^57^ This finding seems to contradict other results that indicate that mammalian cardiomyocytes do not produce functionally relevant amounts of cGMP via NO–sGC.^58^ Recent discoveries using cGMP biosensors for live-cell imaging provide evidence of an intercellular cGMP transfer via gap junctions between murine cardiac fibroblasts and cardiomyocytes.^59^ This previously unrecognized mechanism further expands the numerous functional effects of cGMP to intercellular signalling crosstalk. Intercellular communication was also investigated by Fukuma *et al.*, who recently described a mechanism involving endothelial cells and cardiomyocytes. Oestrogens, through endothelial NO release and in turn cardiomyocyte-specific sGC stimulation, jointly contributed to gender-specific differences in right ventricular remodelling after pulmonary artery banding.^60^

sGC and cell-specific functions of NO–sGC–cGMP signalling in pericytes and (myo)fibroblasts is implicated in the development of fibrosis.^56^ Although sGC is highly expressed by various resident cells in the myocardium,^61^ sGC expression has also been observed in circulating red blood cells (RBCs) of patients both with and without coronary artery disease, with erythrocytes expressing catalytically active sGC (α1β1). Erythrocytes were shown to generate cGMP in response to NO and sGC stimulators, such as BAY 41–2272.^62^ While the extent of sGC signalling in erythrocytes, for instance, has been a matter of debate,^56^ evidence now suggests its involvement in cardioprotection.^63^ A recent study demonstrated that stimulation of the sGC in RBCs obtained from patients with Type 2 diabetes markedly improved cardiac post-ischaemic performance and reduced infarct size when administered to isolated rat hearts. cGMP released following the stimulation of sGC in RBCs acted as a crucial cardioprotective mediator via activation of cardiac PKG.^63^ Moreover, NO–sGC–cGMP signalling controls the regulation of migration, proliferation, differentiation, and endothelial adhesion of leucocytes with an impact on inflammation.^56,64^ In mice, reduced sGC activity worsens cardiac injury and remodelling.^65^ Stimulation of sGC with the experimental sGC stimulator BAY 41-2272 was shown to reduce fibrosis in a hypertensive heart disease rat model.^66^ In both human and murine fibroblasts, BAY 41-2272 inhibited fibrosis by blocking non-canonical TGFβ signalling.^66,67^ Finally, cGMP signalling in human and murine platelets has been implicated in mediating anti-aggregatory and antithrombotic effects.^62,68^

### Potential roles of the NO–sGC–cGMP pathway in chronic heart failure development

Chronic HF is a complex interplay of systemic and local signalling pathways associated with ageing and various comorbidities like hypertension, obesity, and diabetes mellitus. Thus, patients with HF exhibit considerable heterogeneity in their clinical phenotypes. The NO–sGC–cGMP cascade exerts a broad spectrum of effects that may influence the diverse pathophysiological mechanisms and comorbidities associated with HF development. Accordingly, it is not surprising that agents affecting cGMP formation or degradation may exhibit distinct and potentially unique efficacy profiles. Despite the growing body of evidence implicating cGMP as a central mediator in vascular function, fibrosis, and myocardial contractility, the exact mechanisms by which cGMP-dependent pathways influence HF progression remain incompletely understood. Therefore, it is challenging to predict how therapeutic strategies targeting cGMP synthesis or degradation will exert their effects across different stages and phenotypes of HF.

Increased vascular resistance and impaired ventricular relaxation due to decreased NO–sGC–cGMP signalling have been demonstrated in a model of α1-sGC-deficient mice.^14,69,70^ Impaired cardiac contractility arises from a deficiency in cGMP-dependent PKG activity. This leads to decreased phosphorylation of the giant sarcomere protein titin found in cardiomyocytes, resulting in an increase of passive myocardial tension and stiffness.^71–73^ Reduced PKG activity may also accelerate the conversion of cardiac fibroblasts to myofibroblasts, which are known to play a major role in the development of fibrosis and lead to LV stiffness.^73,74^ A study measuring PKG activity and its downstream effects in LV myocardial biopsies reported that patients with HFpEF had lower cGMP concentrations and PKG activity compared with patients with HFrEF or aortic stenosis.^75^ These were associated with increased cardiomyocyte resting tension and were related to higher levels of oxidative stress in patients with HFpEF compared with the other patient populations.^75^ Impaired NO–sGC–cGMP signalling in thoracic aortic tissue lysates, which contain endothelial cells and VSMCs, is implicated in the mechanism underlying vascular inflammation.^14^

cGMP-dependent PKGI (cGKI) is implicated in the development of cardiac fibrosis.^76^ Long-term PKGI activation in mice has been shown to cause hypertrophic remodelling and increased LV dilation and dysfunction, especially in the presence of pressure overload and neurohumoral stress, and to increase cardiac fibrosis with age.^77^ Gain-of-function mutations in *PRKG1*, the gene that encodes PKGI lead to thoracic aortic aneurysms and dissections in mutation-carrying humans.^78,79^ Loss of PKGI activity in cardiac myocytes, in turn, did not lead to changes in cardiac morphology and function under resting conditions in mice. However, angiotensin II or transverse aortic constriction provoked dilated cardiomyopathy with marked deterioration of cardiac function.^80^ A separate study observed that loss of PKGI activity in all non-smooth muscle cells of the heart did not *per se* potentiate the hypertrophic and fibrotic effect of angiotensin II in mice.^81^ The PDE5 inhibitor, sildenafil, in turn was shown to attenuate fibrosis but had no effect on hypertrophy in this model,^81^ which suggests that the antifibrotic actions of this PDE5 inhibitor must depend on a (cardiac) PDE5/cGMP/PKGI pathway in at least one cell type other than smooth muscle cells. Indeed, although sildenafil was not directly tested, more recent evidence implies that PKGI deficiency confined to *periostin*-positive cardiac myofibroblasts exacerbates the angiotensin II-induced structural and morphological remodelling processes in the mouse heart.^82^ Mixed lineage kinase 3 (MLK3) has been identified as a PKGIα substrate, presumably in cardiac myocytes, that is required for *in vivo* therapeutic effects of PKGI activation with sildenafil after pressure overload. This finding, especially regarding the effect of sildenafil on the PKGI–MLK3 axis, is somewhat surprising, albeit MLK3 also plays a role in the control of blood pressure and vascular stiffness independently of PKGIα.^83^ Together, these results underline the potential importance of developing an appropriate spatially and temporally co-ordinated treatment strategy through cGMP/PKGI in HF.

Disruption of the NO–sGC–cGMP pathway is a key mechanism in the development of atherosclerosis,^13,27^ an inflammatory disorder that can result in CAD, a major risk factor for the development of HFrEF. Reduced NO bioavailability as a result of oxidative stress decreases endothelial NO regulation of, for example, leucocyte adhesion and monocyte migration, which are key early steps in the development of atherosclerosis.^11,64^ sGC expression was reduced in aortic tissue in an animal model of atherosclerotic CV disease,^84^ and loss of α1-sGC in mice is associated with an elevated risk of MI, presumably due to accelerated thrombus formation.^30^ nNOS-derived NO facilitates cardiac relaxation, thereby regulating cardiac contractility and rhythm.^85^  *In vivo* animal studies show that the expression and activity of nNOS are increased following cardiac injury and early in HF.^85^ The resulting increase in nNOS-derived NO has cardioprotective benefits, including a reduction in LV dilation, hypertrophy and infarct size, and decreased risk of arrhythmias.^85^

Altered calcium ion handling and electrophysiology are features of HF aligned with diastolic dysfunction and arrhythmias. Atrial fibrillation is commonly associated with HF, and each condition begets the other.^86^ Reduced production of cGMP impairs normal function of cGKI, an important modulator of intracellular calcium levels.^42^ Increased intracellular calcium concentrations in human atrial myocytes due to diastolic leakage from ryanodine receptors increase after depolarizations^87,88^; in animal models, these pathways have been shown to activate profibrotic pathways leading to structural remodelling that promotes atrial fibrillation.^89,90^ The resulting loss of atrial contraction impacts ventricular calcium ion cycling and reduces cardiac output, thereby exacerbating HF.^86^

## Pharmacological modulation of the NO–sGC–cGMP pathway

There are several pharmacological tools targeting key elements of the NO–sGC–cGMP pathway, including NO and cGMP formation, and cGMP degradation by PDEs.

NO donors have been in clinical use for over a century for the alleviation of the symptoms of angina pectoris.^35^ NO donors (e.g. nitrates), which release NO and subsequently trigger NO-binding and activation of sGC, have been shown to have beneficial effects on LV function and exercise capacity when used in combination with other antihypertensive drugs.^91^ Activation of organic nitrates, sodium nitroprusside, and S-nitrosothiols occurs enzymatically.^35,92^ S-nitrosothiols also release NO in response to factors including light, heat, superoxide, and transition metals, and diazeniumdiolates decompose spontaneously.^92^ These non-enzymatic mechanisms of NO release result in a lower tolerance potential of these agents.^92^ The liberated NO binds to sGC, resulting in the production of cGMP.^35,92^ However, poor tolerability of adverse effects, formation of free radicals, restricted bioavailability, and undesirable off-target effects limit the selectivity of nitrates and their effectiveness long-term.^35,92,93^ Hydrogen sulphide (H_2_S) has also gained recognition as a facilitator of NO signalling.^38^ In addition to increasing the production of NO, H_2_S has been shown to inhibit the degradation of cGMP and to facilitate PKG activation.^38^

Another interesting approach for increasing NO that is under discussion is the use of dietary supplementation of inorganic nitrate in the form of beetroot juice or extract; however, there is currently insufficient evidence of the CV benefits of this approach.^94,95^

Besides direct NO supplementation, the levels of cGMP can be increased by blocking the degradation of cGMP by inhibition of cGMP-degrading PDEs. The first available PDE5 inhibitors were introduced for the treatment of erectile dysfunction (ED).^96–98^ The first compound approved for ED treatment was sildenafil in 1998, followed by tadalafil (2002 in Europe and 2003 in the USA) and vardenafil (2003 in both Europe and the USA).^96–100^ Sildenafil and tadalafil were also approved for the treatment of PAH in 2005 and 2008, respectively.^101,102^ The efficacy of PDE5 inhibitors for both applications is due to the vascular action of PDE5 inhibition, thereby stabilizing cGMP-induced vasorelaxation in penile and lung vascular beds.^96–98,101,102^ However, not all patients with either ED or PAH respond adequately to PDE5 inhibition.^103–108^ This could be explained mechanistically by a lack of sufficient endogenous cGMP production, mainly due to endothelial dysfunction and the resulting NO depletion due to ageing.^109^ It may also be driven by prevalent comorbidities in patients with HF, such as metabolic syndrome, dyslipidaemia, obesity or diabetes.^110–113^

Sildenafil demonstrated cardioprotective effects in preclinical models of pressure overload and ischaemia–reperfusion injury, reducing heart chamber and myocyte hypertrophy.^76,114^ Its effects were initially attributed to modification of PKGI function in cardiomyoctes.^76^ A lack of benefit in clinical studies of patients with HF^21,22,115,116^ prompted a re-evaluation of these findings, and it is now thought likely that cell types other than cardiomyocytes account for the majority of PDE5 activity in the failing heart.^76^

PDE9 inhibitors are currently not available for clinical use; selective and potent PDE9 inhibitors have been identified in the past and have been evaluated in preclinical profiling and non-clinical models.^117,118^ Therefore, the clinical relevance of non-clinical reports of CV effects of PDE9 inhibitors, e.g. suppression of heart hypertrophy,^119^ is still missing translation.

### Mode of action of NO-independent and haem-(in)dependent soluble guanylyl cyclase modulators

Given the limitations of NO-donors and PDE5 inhibitors, non-small molecules that do not liberate NO but which can bind to sGC directly and stimulate the enzyme independently of NO were identified. Such sGC stimulators were first discovered in 1994 using platelets.^120^ NO-independent direct stimulation of sGC to increase intracellular cGMP levels was verified in 1997 using VSMCs, and the term ‘stimulator of soluble guanylyl cyclase’ was used in a publication by Mülsch *et al.*^121^ These first haem-dependent sGC stimulators had several shortcomings including poor stability, light sensitivity, weak sGC-stimulating potency, highly variable pharmacokinetic profiles, and lack of specificity, and they were not suitable for clinical use.^35^ Structure-based approaches and screening commenced to identify molecules with the same molecular mode of action but with improved physicochemical properties that could be used clinically, leading to the discovery of riociguat.^35^

**Figure 2:**
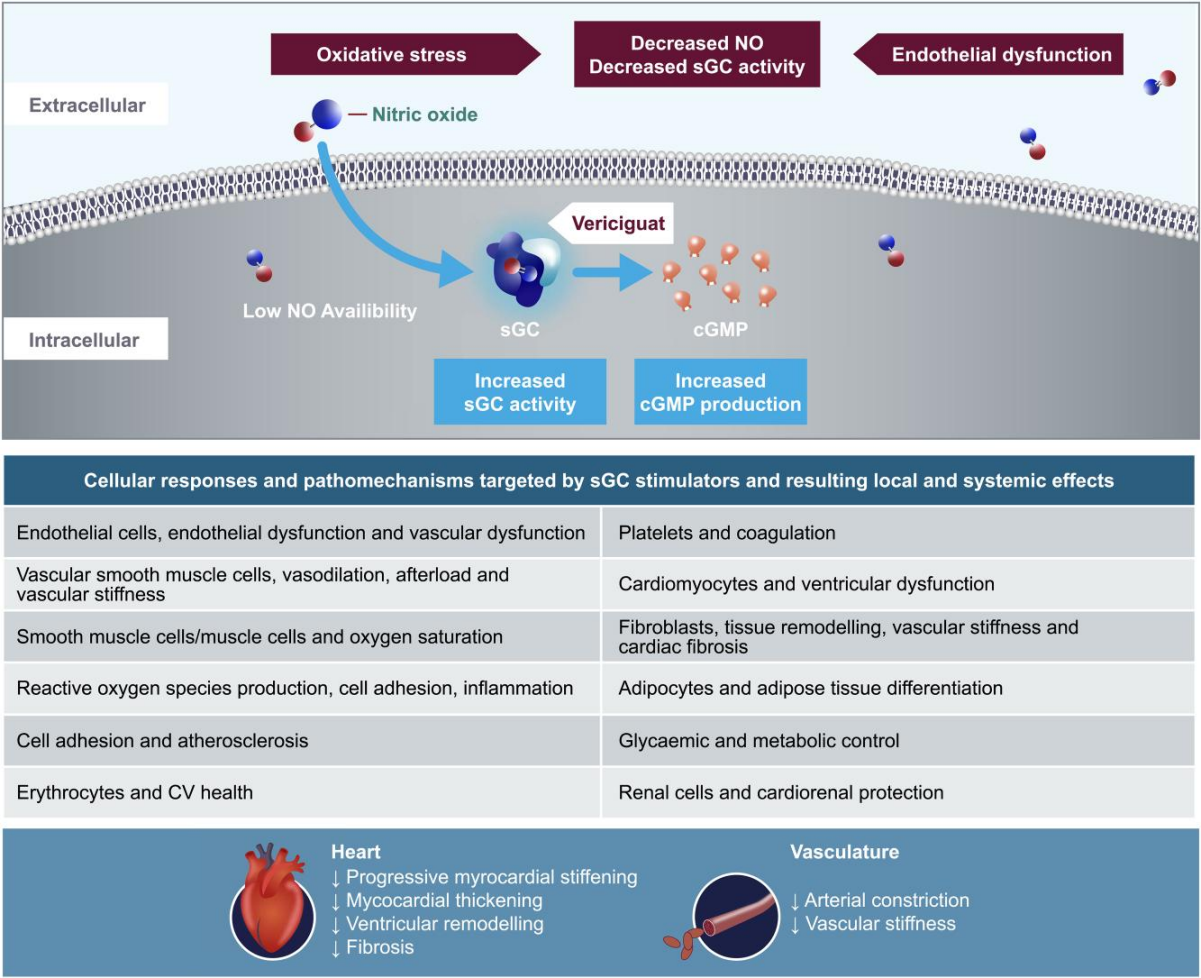
Dual mechanism of action of vericiguat on the pathomechanisms associated with HF. Vericiguat is an oral sGC stimulator. It has a dual mechanism of action: it increases cGMP independent of NO by allosteric binding to the NO-binding site and sensitizes sGC to endogenous NO by stabilizing the NO–sGC binding. Vericiguat treatment is expected to restore the impaired NO–sGC–cGMP pathway in HF, leading to a diversity of pharmacological effects including improvements in cardiac and vascular functions, and reduction in profibrotic and inflammatory pathways thus reducing adverse clinical outcomes in patients with worsening chronic HFrEF. Refer to the main text for details of experimental models used to investigate pathomechanisms. CV, cardiovascular; cGMP, cyclic guanosine monophosphate; HF, heart failure; HRrEF, heart failure with reduced ejection fraction; NO, nitric oxide; sGC, soluble guanylyl cyclase

Riociguat, the first sGC stimulator approved for clinical use, is indicated for the treatment of chronic thromboembolic pulmonary hypertension and PAH. However, it has little efficacy in HF, and its use in HF is further complicated by its several drug interactions and its short half-life, which requires a thrice-daily treatment regimen.^122,123^ Optimization of the pharmacokinetic profile of sGC stimulators led to the discovery of vericiguat, which was approved for the treatment of patients with HFrEF in 2021.^122^ Riociguat and vericiguat stimulate sGC independently of NO and have effects that are synergistic with endogenous NO in low and normal NO environments (*Figure 2*). The activity of these agents is dependent on the native haem,^92^ with sGC stimulators stabilizing the NO binding to the haem-group.^35,92^ Besides sGC stimulators, a second class of compounds with a different molecular mode of action was discovered, sGC activators, which activate oxidized and haem-free sGC (*Figure 1*) and are haem-independent.^35^ The redox equilibrium between native sGC and its oxidized or haem-deficient forms remains incompletely understood. Moreover, the specific diseases and conditions under which sGC stimulators or activators are most effective continue to be subjects of ongoing debate. This should be an avenue of further research, since sGC activators have advanced through clinical development in chronic kidney disease and might also be available for patients with HF in future.

## Cellular responses and pathomechanisms targeted by soluble guanylyl cyclase stimulators and the resulting local and systemic effects—preclinical data

HF is not a single disease, but rather a complex clinical syndrome encompassing a heterogeneous array of underlying pathologies. Given the multifactorial and highly heterogeneous nature of HF, the precise mechanisms of action of current standard therapies—such as ARNi, BBs, MRAs, and SGLT2is—at the cellular and tissue levels, as well as their systemic benefits, remain poorly understood.^124^ In this context, vericiguat, which was added to the recommended medical therapy for worsening HFrEF,^125^ is no exception. As for other standard of care therapies, the mode of action and relative contributions of vericiguat that result in its beneficial outcomes are only partly understood, and basic research is needed to clarify the cellular responses to sGC stimulation. Nevertheless, there are several pathomechanisms leading to chronic HF in which cGMP increase and sGC stimulation have proven to be effective in preclinical studies. Key pathomechanisms are described here, while other mechanisms and cellular responses, involving VSMCs, ROS production, cell adhesion, platelets, and adipocytes are described in Part 1 of the Supplementary material.

### Endothelial cells, endothelial dysfunction, and vascular dysfunction

In chronic HF, a pro-inflammatory state promotes vascular endothelial dysfunction, leading to excess production of endothelium-derived reactive oxygen species (ROS) and reactive nitrogen species.^12,27^ This results in oxidative stress and an imbalance of NO and ROS, which leads to reduced NO bioavailability.^11–13^ Oxidative stress induces cell proliferation, hypertrophy, apoptosis, and inflammation via several mechanisms.^27^ The oxidative stress renders sGC unresponsive to NO,^13^ which results in disruption of the NO–sGC–cGMP signalling cascade and impairs endothelium-dependent vasodilation within the coronary vasculature.^27^ In humans, impaired vasodilation promotes vascular stiffness, adverse remodelling and fibrosis, and decreases coronary blood flow, leading to cardiac dysfunction.^11,12,61,126–128^ In addition, two clinical trials in patients with HF and/or coronary heart disease demonstrated that dysfunctional vasodilation led to systemic microvascular dysfunction.^129,130^ It is well established that sGC stimulators, including vericiguat, are also able to dilate blood vessels and coronary arteries under conditions of very low endogenous NO-production or even in the absence of NO.^131^ This has been demonstrated in different vascular beds including coronary arteries and peripheral blood vessels.^131^ Thus, vericiguat is able to exert both a local dilatory effect on the myocardium as well as systemic vasodilation. The former might improve microperfusion and cardiac function, while the latter could help to unload the heart.

### Erythrocytes and cardiovascular health

NO–sGC–cGMP signalling in RBCs has gained a lot of interest in recent years, and intact NO–sGC–cGMP signalling in RBCs has been shown to be cardioprotective.^132,133^

Human and murine RBCs carry catalytically active sGC-α1, PDE5, and PKG.^62^ Mouse RBCs exposed to hypoxia demonstrated sGC-dependent increased extracellular export of cGMP, which improved post-ischaemic cardiac function and reduced infarct size when applied to isolated mouse hearts at the onset of ischaemia.^133^ Oral administration of nitrate to mice enhanced the cardioprotective effect of hypoxic RBCs.^133^ RBCs from nitrate-treated subjects were also shown to be cardioprotective in isolated rat hearts subjected to ischaemia reperfusion, significantly improving post-ischaemic cardiac recovery compared with RBCs from subjects with low nitrate intake.^133^ Pre-incubation of the RBCs from the nitrate-fed subjects with an sGC inhibitor abolished the effects on the rat heart, suggesting a key role of sGC in the cardioprotective effects of RBCs.^133^ A further study showed that sGC stimulation of RBCs from individuals with Type 2 diabetes attenuated impaired cardiac function after ischaemia–reperfusion injury in isolated rat hearts.^63^

While eNOS expression in RBCs was shown to be decreased in patients with stable CAD and endothelial dysfunction compared with age-matched controls, sGC responsiveness to NO was maintained.^62^

In a placebo-controlled study with the sGC stimulator riociguat in participants with sickle cell disease and stage 1 hypertension or proteinuria (*n* = 130), the rate of sickle cell-related vaso-occlusive events was similar between treatments.^134^

### Cardiomyocytes and ventricular dysfunction

Myocardial hypertrophy initially serves a compensatory role in HF by mitigating wall stress and normalizing oxygen demand during ventricular haemodynamic overload, thereby preserving systolic function. However, during HF progression and worsening, hypertrophy causes a decline of cardiac function. Thus, antihypertrophic efficacy could be beneficial in HF. Preclinical studies with several sGC stimulators (vericiguat, riociguat, BAY 41-2272 and BAY 41-8543) have demonstrated beneficial actions on the heart, including anti-hypertrophic and anti-proliferative effects on cardiomyocytes^135^ and myofibroblasts,^66,136^ resulting in a range of pharmacological effects such as reduced cardiac hypertrophy.^122,137,138^

However, cardiomyocyte-specific PKG knockout mice develop the same degree of cardiomyocyte hypertrophy as wild-type mice.^139^  *In vitro* and *in vivo* studies have shown that cardiomyocytes lack NO-induced cGMP signalling but that cGMP formed in fibroblasts in response to NO enters cardiomyocytes via gap junctions and stimulates downstream effects.^140^ Whether cGMP increase exerts a direct or indirect antihypertrophic effect on cardiomyocytes remains unresolved. However, numerous *in vivo* studies have consistently demonstrated a reduction in heart hypertrophy, as assessed by heart-to-body weight ratios, with the use of sGC stimulators, including vericiguat.^122,137,138,141^

### Fibroblasts, tissue remodelling, vascular stiffness, and cardiac fibrosis

In addition to their effects on endothelial dysfunction, ROS production, and inflammation, sGC stimulators may also directly modulate fibrosis through cGMP-mediated pathways. NO-induced cGMP signalling has been demonstrated in isolates of murine cardiac fibroblasts,^140^ and the sGC stimulators, BAY 41-2272 and vericiguat, have been shown to inhibit fibroblast-to-myofibroblast differentiation^67^ and also to decrease collagen production.^66,67,142^ In several non-clinical studies, an anti-fibrotic effect was observed with the sGC stimulator, BAY41-2272, in hypertensive rats, including reducing both interstitial and perivascular heart fibrosis.^66^ Notably, these effects were independent of blood pressure reduction.^66,136^ sGC stimulation with riociguat reduced myocardial fibrosis and a myocyte cross-sectional area in mice with transverse aortic constriction-induced HF and cardiac remodelling.^137^

### Renal cells and cardiorenal protection

In a hypertensive kidney failure model, the sGC stimulator vericiguat led to a decrease of proteinuria, indicating a potential kidney protective effect.^122^ Praliciguat reduced proteinuria in a rat model of hypertension, suggesting that this effect might be at least partially related to the blood-pressure-lowering effect of sGC stimulators.^143^ Suppression of inflammation and apoptosis in human renal proximal tubular cells observed with praliciguat indicates that tubular mechanisms may also contribute to its kidney effects.^144^

These different modes of action, describing how increased cGMP and sGC stimulation can be beneficial in CV disease and HF, come from basic pharmacological knowledge on the cellular effects and responses to sGC stimulation and cGMP. This evidence, even if yet incomplete, may direct our understanding of the mechanisms by which vericiguat may contribute to the therapeutic effects in the complex pathophysiology of chronic HF. In most cases, these data from preclinical mechanistic studies and disease models require clinical validation to confirm their efficacy and relevance in populations of patients with HF and even subpopulations of patients with HF. This is especially important for understanding the full therapeutic potential of vericiguat in HF, and for quantifying the specific contributions of each distinct mode of action. In the following sections, the clinical data with vericiguat are summarized.

In summary, there is robust evidence from non-clinical *in vitro*, *ex vivo*, and *in vivo* studies which imply a broad mode of action spectrum of sGC stimulation in HF. However, clinical information pertaining to these different mechanisms and their contributions to the overall efficacy remains sparse.

## Clinical profile of vericiguat and clinical study update

Vericiguat is indicated in the EU, USA, and other countries for the treatment of symptomatic chronic HF following a worsening event in adult patients with HFrEF.^25,145^ It has been recognized in recent regional guidelines for use in patients with worsening HFrEF^5,7^ after demonstrating benefits in patients with HFrEF in two pivotal trials: the Phase 2 SOCRATES-REDUCED trial and the Phase 3 VICTORIA trial (*Table 1*).^146,148^ A third trial, VICTOR, was conducted to extend the available data on the efficacy and tolerability of vericiguat to patients with HF without a recent worsening/decompensation event (*Table 1*).^150^

**Table 1:** Design and key findings of randomized, controlled trials with vericiguat in patients with HFrEF

	SOCRATES-REDUCED (NCT01951625)^146,147^	VICTORIA (NCT02861534)^148,149^	VICTOR (NCT05093933)^150^
**Phase**	2	3	3
**Number of enrolled patients**	456	5050	6105
**Design**	Randomized parallel-group, placebo-controlled, double-blind, multicentre dose-finding	Randomized, placebo-controlled, parallel-group, multicentre, double-blind	Randomized, placebo-controlled, parallel-group, multicentre, double-blind
**Vericiguat regimen**	1.25 mg, 2.5 mg, 5 mg or 10 mg orally once daily (target doses). All vericiguat groups, (except 1.25 mg) started on 2.5 mg and were titrated up to the target dose	Starting dose of 2.5 mg, orally once daily titrated to 5 mg and to a target dose of 10 mg	Starting dose of 2.5 mg, orally once daily titrated to 5 mg and to a target dose of 10 mg
**Patient population**	Chronic HFrEF (NYHA class II to IV; EF <45%) Requiring HHF (or intravenous diuretic treatment for HF without hospitalization) with initiation of study treatment after clinical stabilization On GDMT Male and female Age ≥18 years Elevated NT-proBNP (baseline median: 3076 pg/mL)	Chronic HFrEF (NYHA class II to IV; EF <45%) HHF within 6 months or the need for outpatient intravenous diuretics within 3 months before randomization On GDMT Male and female Age ≥18 years Elevated NT-proBNP (baseline median: 2816 pg/mL)	Chronic HFrEF (NYHA class II to IV; EF ≤40%) No HHF within 6 months or the need for outpatient intravenous diuretics within 3 months before randomization On GDMT Male and female Age ≥18 years Elevated NT-proBNP (baseline median: 1375 pg/mL)
**Primary endpoint**	Change in log-transformed NT-proBNP from baseline to week 12	Composite of CV death or HHF	Composite of CV death or HHF
**Duration of follow-up**	16 weeks	Median 10.8 months	18.5 months
**Key findings**	Change in NT-proBNP at week 12: no significant difference between the pooled vericiguat group and placebo (difference of means, −0.122; 90% CI, −0.32 to 0.07; ratio of geometric means, 0.885, 90% CI, 0.73–1.08; *P* = .15).^a^ Dose-response relationship demonstrating higher vericiguat doses were associated with greater reductions in NT-proBNP level (*P* < .02)	Primary endpoint occurred in 37.8% of vericiguat group, and 40.9% of the placebo group (HR, 0.90; 95% CI, 0.83–0.98; *P* = .02)	Primary endpoint occurred in 18.0% of the vericiguat group, and 19.1% of the placebo group (HR, 0.93; 95% CI, 0.83–1.04; *P* = .22).^a^ CV death occurred in 9.6% of the vericiguat group and 11.3% of the placebo group (HR, 0.83; 95% CI, 0.71–0.97). HHF occurred in 11.4% of the vericiguat group and 11.9% of the placebo group (HR, 0.95; 95% CI, 0.82–1.10)

CI, confidence interval; CV, cardiovascular; EF, ejection fraction; GDMT, guideline-directed medical therapy; HF, heart failure; HFrEF, heart failure with reduced ejection fraction; HHF, hospitalization for heart failure; HR, hazard ratio; NT-proBNP, N-terminal pro-B-type natriuretic peptide; NYHA, New York Heart Association.

^a^Denotes trials for which the primary endpoint was neutral.

The dose-finding SOCRATES-REDUCED study (*n* = 456; *Table 1*) evaluated the change in the level of N-terminal pro-B-type natriuretic peptide (NT-proBNP) from baseline to week 12 with vericiguat (1.25, 2.5, 5, and 10 mg) compared with placebo in patients with worsening HFrEF.^146^ Change in NT-proBNP, a prognostic biomarker for HF, did not differ significantly between vericiguat (pooled group) and placebo; however, higher doses of vericiguat were associated with greater reductions in NT-proBNP (*P* < .02).^146^

In the VICTORIA study (*n* = 5050), vericiguat significantly reduced the composite primary outcome of death from CV causes or hospitalization for HF (HHF) compared with placebo.^148^ There was no significant reduction in either all-cause or CV mortality. Specifically, vericiguat decreased the residual risk of CV death or HHF from 37.8 per 100 patient-years to 33.6 per 100 patient-years.^148^ Based on an absolute reduction of 4.2 events per 100 patient years, the number needed to treat with vericiguat for 1 year to prevent a primary outcome event is approximately 24 patients.^148^ A reduction in the primary composite endpoint was observed in patients with an NT-proBNP level up to 8000 pg/ml.^151^

Subgroup analyses of VICTORIA have demonstrated that the benefit of vericiguat on the primary composite endpoint was independent of age,^152^ sex,^152^ baseline LVEF,^153^ comorbid CAD,^154^ baseline systolic blood pressure,^155^ baseline kidney function,^156^ and background therapy.^157,158^

In VICTORIA, vericiguat was administered in addition to guideline-directed medical therapy (GDMT), which was well balanced between the vericiguat and placebo groups.^148^ A subset of 731 patients received ARNi at randomization,^159^ and concomitant neprilysin inhibition with ARNi did not alter the efficacy of vericiguat in patients enrolled in the trial.^159^ Based on the results from the VICTORIA study, vericiguat may be considered for the treatment of patients with HFrEF already receiving GDMT (European Society of Cardiology guideline recommendations (2021): Class IIb, Level of evidence B; American Heart Association/American College of Cardiology/Heart Failure Society of America guideline recommendations (2022): Class 2b, Level of evidence B-R).^5,7^

The proportion of adverse events (serious and non-serious) that occurred in the vericiguat group in VICTORIA was similar to that in the placebo group.^148^ Prespecified safety events of clinical interest were symptomatic hypotension and syncope. Symptomatic hypotension occurred in 9.1% of the patients in the vericiguat group vs 7.9% of the patients in the placebo group (*P* = .12). Syncope occurred in 4.0% of patients in the vericiguat group and in 3.5% of patients in the placebo group (*P* = .30).^148^ No excessive reductions in blood pressure were observed with vericiguat in subgroups of patients in VICTORIA potentially predisposed to hypotension, such as older patients, those with lower baseline blood pressure, and patients receiving concurrent ARNi therapy.^155^ In a separate dedicated QTc study, vericiguat demonstrated a low potential for cardiac arrhythmias,^160^ which is an associated risk for patients with worsening HF. Rates of hyperkalaemia and worsening renal function, and serum potassium and renal function trajectories in VICTORIA did not differ between the vericiguat and placebo groups.^156^

The Phase 3 VICTOR study (*n* = 6105) evaluated the efficacy and safety of vericiguat in adult patients with chronic HFrEF with no HHF within 6 months or outpatient intravenous diuretic use within 3 months before randomization (*Table 1*).^150^ The primary composite endpoint of time to CV death or first HHF was not statistically significant between the vericiguat (18.0%) and placebo (19.1%) groups (*P* = .22).^150^ The most common adverse event in VICTOR was symptomatic hypotension (vericiguat: 11.3%; placebo: 9.2%).^150^

VICTOR was designed and powered to assess the effect of vericiguat on CV mortality, which was conditional on the primary endpoint success. Although vericiguat was neutral for the primary composite endpoint of CV death or HHF in ambulatory well-treated participants with HFrEF, a numerical reduction in CV death, favouring vericiguat was observed, with a hazard ratio (HR) of 0.83 (95% confidence interval [CI] 0.71–0.97); nominal *P* = .02).^161^ Reductions in all-cause mortality, sudden cardiac death and HF-related death were also observed. However, all secondary endpoint results should be interpreted cautiously as the *P* values are nominal.

Discrepancies in efficacy outcomes between the VICTORIA and VICTOR trials likely reflect differences in patient populations between the two studies. While patients with baseline NT-proBNP concentrations >6000 pg/mL (i.e. those with more advanced disease) were excluded from VICTOR, there was no upper limit for baseline NT-proBNP levels in patients in VICTORIA.^148^ In addition, in contrast to VICTORIA, VICTOR included a contemporary GDMT-treated population of ambulatory patients, which included use of ARNi and SGLT2i in a large proportion, and excluded those with recent worsening HF.^150^

An exploratory prespecified, pooled individual participant-level analysis of the VICTORIA and VICTOR trials (*n* = 11 155) included patients with HFrEF across a broad range of clinical severity. Treatment with vericiguat reduced the primary composite endpoint of CV death or HHF (vericiguat: 25.9%; placebo: 27.9%; HR 0.91 [95% CI 0.85–0.98]; *P* = .009]), with similar reductions in CV death (HR 0.89 [95% CI 0.80–0.98]; *P* = .02) and HHF (HR 0.92 [95% CI 0.84–1.00]; *P* = .04) as first events.^162^ Interpretation of these pooled analysis data should be made cautiously owing to between-trial differences, such as different baseline NT-proBNP levels, treatment eras and background therapy.^163^ It is anticipated that the pooled analysis findings will guide patient selection in future use of the drug.

Despite clinical improvements in HFrEF, the efficacy of vericiguat has not been established in HFpEF; further details on key clinical trials in HFpEF are provided in Supplementary material, Part 2.

### Research gaps and future directions

The risk of adverse outcomes varies over time in patients with HFrEF, with many patients experiencing episodes of worsening HF following periods of relative stability.^164^ Tailoring treatment according to the underlying risk profile is important for optimizing the management of HF.

Current GDMT for HFrEF comprises a combination of up to four substances (ACEi/ARB/ARNi, a BB, an MRA, and an SGLT2i) uptitrated to maximally tolerated doses, with rapid sequential or simultaneous initiation recommended following a worsening HF event.^5,7^ Existing data suggest that vericiguat may be considered for adjunctive treatment, alongside current GDMT, in patients with HFrEF who have had worsening (i.e. decompensation of HF). Further clinical studies are needed to determine whether early, simultaneous use of vericiguat, alongside optimized GDMT, might be effective in HF.

There remain unanswered mechanistic questions that could inform the development of other agents acting on the pathway, including: the role of cGMP compartmentalization, interplay with natriuretic peptide/cGMP signalling, differential effects in HFpEF vs HFrEF, and the impact of high-dose SGLT2i/ARNi.

Additional areas of interest include the investigation of the potential of vericiguat in patients with comorbidities associated with dysfunctional NO–sGC–cGMP signalling, such as pulmonary hypertension and chronic kidney disease.^165^ Further potential avenues of research include identifying factors that may modify the treatment effect of sGC stimulators, such as the duration of HF, history of HHF, recent volume overload and the underlying aetiologies of HFrEF. Pharmacological therapies that affect the NO–sGC–cGMP pathway but have not been indicated as a therapy for HF are described in Part 2 of the Supplementary section.

## Summary and outlook

Ascertaining the clinical benefit of pharmacological interventions targeting and enhancing cGMP signalling in HF has been challenging. We are just beginning to understand the mode of action pathway utilized by sCG stimulators and the response of the relevant cells and tissues to these medications. By acting in a complementary manner to current GDMT, sGC stimulators have the potential to provide clinical benefits for patients at risk of further worsening of HF. Further studies are needed to determine how the use of these agents may be optimized in clinical practice.

## Supplementary Material

xvag117_Supplementary_Data

## Declarations

### Data Availability

No data were generated or analysed for this manuscript.

## References

[1] Savarese G, Becher PM, Lund LH, Seferovic P, Rosano GMC, Coats AJS. Global burden of heart failure: a comprehensive and updated review of epidemiology. Cardiovasc Res 2023;118:3272–87. 10.1093/cvr/cvac013 35150240

[2] Ruiz-Garcia A, Serrano-Cumplido A, Escobar-Cervantes C, Arranz-Martinez E, Turegano-Yedro M, Pallares-Carratala V. Heart failure prevalence rates and its association with other cardiovascular diseases and chronic kidney disease: SIMETAP-HF study. J Clin Med 2023;12:4924. 10.3390/jcm12154924 37568326 PMC10419820

[3] Zhang L, Ono Y, Qiao Q, Nagai T. Trends in heart failure prevalence in Japan 2014-2019: a report from healthcare administration databases. ESC Heart Fail 2023;10:1996–2009. 10.1002/ehf2.14321 37016908 PMC10192231

[4] Bellanca L, Linden S, Farmer R. Incidence and prevalence of heart failure in England: a descriptive analysis of linked primary and secondary care data—the PULSE study. BMC Cardiovasc Disord 2023;23:374. 10.1186/s12872-023-03337-1 37495953 PMC10373419

[5] McDonagh TA, Metra M, Adamo M, Gardner RS, Baumbach A, Böhm M, et al 2021 ESC guidelines for the diagnosis and treatment of acute and chronic heart failure: developed by the Task Force for the diagnosis and treatment of acute and chronic heart failure of the European Society of Cardiology (ESC) with the special contribution of the Heart Failure Association (HFA) of the ESC. Eur Heart J 2021;42:3599–726. 10.1093/eurheartj/ehab368 34447992

[6] McDonagh TA, Metra M, Adamo M, Gardner RS, Baumbach A, Böhm M, et al 2023 Focused update of the 2021 ESC guidelines for the diagnosis and treatment of acute and chronic heart failure. Eur Heart J 2023;44:3627–39. 10.1093/eurheartj/ehad195 37622666

[7] Heidenreich PA, Bozkurt B, Aguilar D, Allen LA, Byun JJ, Colvin MM, et al 2022 AHA/ACC/HFSA guideline for the management of heart failure: a report of the American College of Cardiology/American Heart Association Joint Committee on Clinical Practice Guidelines. Circulation 2022;145:e895–1032. 10.1161/cir.0000000000001063 35363499

[8] Schiattarella GG, Rodolico D, Hill JA. Metabolic inflammation in heart failure with preserved ejection fraction. Cardiovasc Res 2021;117:423–34. 10.1093/cvr/cvaa217 32666082 PMC8599724

[9] Anker SD, Butler J, Filippatos G, Ferreira JP, Bocchi E, Bohm M, et al Empagliflozin in heart failure with a preserved ejection fraction. N Engl J Med 2021;385:1451–61. 10.1056/NEJMoa2107038 34449189

[10] Solomon SD, McMurray JJV, Claggett B, de Boer RA, DeMets D, Hernandez AF, et al Dapagliflozin in heart failure with mildly reduced or preserved ejection fraction. N Engl J Med 2022;387:1089–98. 10.1056/NEJMoa2206286 36027570

[11] Gheorghiade M, Marti CN, Sabbah HN, Roessig L, Greene SJ, Bohm M, et al Soluble guanylate cyclase: a potential therapeutic target for heart failure. Heart Fail Rev 2013;18:123–34. 10.1007/s10741-012-9323-1 22622468

[12] Greene SJ, Gheorghiade M, Borlaug BA, Pieske B, Vaduganathan M, Burnett JC Jr, et al The cGMP signaling pathway as a therapeutic target in heart failure with preserved ejection fraction. J Am Heart Assoc 2013;2:e000536. 10.1161/JAHA.113.000536 24334823 PMC3886746

[13] Stasch JP, Pacher P, Evgenov OV. Soluble guanylate cyclase as an emerging therapeutic target in cardiopulmonary disease. Circulation 2011;123:2263–73. 10.1161/circulationaha.110.981738 21606405 PMC3103045

[14] Rizzo NO, Maloney E, Pham M, Luttrell I, Wessells H, Tateya S, et al Reduced NO-cGMP signaling contributes to vascular inflammation and insulin resistance induced by high-fat feeding. Arterioscler Thromb Vasc Biol 2010;30:758–65. 10.1161/ATVBAHA.109.199893 20093624 PMC2865555

[15] Calderone A, Thaik CM, Takahashi N, Chang DL, Colucci WS. Nitric oxide, atrial natriuretic peptide, and cyclic GMP inhibit the growth-promoting effects of norepinephrine in cardiac myocytes and fibroblasts. J Clin Invest 1998;101:812–8. 10.1172/jci119883 9466976 PMC508629

[16] SoRelle R . Nobel prize awarded to scientists for nitric oxide discoveries. Circulation 1998;98:2365–6. 10.1161/01.cir.98.22.2365 9832478

[17] Billing AM, Kim YC, Gullaksen S, Schrage B, Raabe J, Hutzfeldt A, et al Metabolic communication by SGLT2 inhibition. Circulation 2024;149:860–84. 10.1161/CIRCULATIONAHA.123.065517 38152989 PMC10922673

[18] Zhang N, Feng B, Ma X, Sun K, Xu G, Zhou Y. Dapagliflozin improves left ventricular remodeling and aorta sympathetic tone in a pig model of heart failure with preserved ejection fraction. Cardiovasc Diabetol 2019;18:107. 10.1186/s12933-019-0914-1 31429767 PMC6702744

[19] Zhuang XD, Long M, Li F, Hu X, Liao XX, Du ZM. PDE5 inhibitor sildenafil in the treatment of heart failure: a meta-analysis of randomized controlled trials. Int J Cardiol 2014;172:581–7. 10.1016/j.ijcard.2014.01.102 24534379

[20] Hwang IC, Kim YJ, Park JB, Yoon YE, Lee SP, Kim HK, et al Pulmonary hemodynamics and effects of phosphodiesterase type 5 inhibition in heart failure: a meta-analysis of randomized trials. BMC Cardiovasc Disord 2017;17:150. 10.1186/s12872-017-0576-4 28606099 PMC5468951

[21] Liu LC, Hummel YM, van der Meer P, Berger RM, Damman K, van Veldhuisen DJ, et al Effects of sildenafil on cardiac structure and function, cardiopulmonary exercise testing and health-related quality of life measures in heart failure patients with preserved ejection fraction and pulmonary hypertension. Eur J Heart Fail 2017;19:116–25. 10.1002/ejhf.662 27873388

[22] Redfield MM, Chen HH, Borlaug BA, Semigran MJ, Lee KL, Lewis G, et al Effect of phosphodiesterase-5 inhibition on exercise capacity and clinical status in heart failure with preserved ejection fraction: a randomized clinical trial. JAMA 2013;309:1268–77. 10.1001/jama.2013.2024 23478662 PMC3835156

[23] Guay CA, Morin-Thibault LV, Bonnet S, Lacasse Y, Lambert C, Lega JC, et al Pulmonary hypertension-targeted therapies in heart failure: a systematic review and meta-analysis. PLoS One 2018;13:e0204610. 10.1371/journal.pone.0204610 30307953 PMC6181322

[24] Hussain I, Mohammed SF, Forfia PR, Lewis GD, Borlaug BA, Gallup DS, et al Impaired right ventricular-pulmonary arterial coupling and effect of sildenafil in heart failure with preserved ejection fraction: an ancillary analysis from the phosphodiesterase-5 inhibition to improve clinical status and exercise capacity in diastolic heart failure (RELAX) trial. Circ Heart Fail 2016;9:e002729. 10.1161/circheartfailure.115.002729 27072860 PMC4831074

[25] Food and Drug Administration . *VERQUVO Prescribing Information*. 2021. https://www.accessdata.fda.gov/drugsatfda_docs/label/2021/214377s000lbl.pdf (8 February 2021, date last accessed).

[26] European Medicines Agency . *Verquvo Summary of Product Characteristics*. 2021. https://www.ema.europa.eu/en/documents/product-information/verquvo-epar-product-information_en.pdf (31 July 2023, date last accessed).

[27] Higashi Y, Noma K, Yoshizumi M, Kihara Y. Endothelial function and oxidative stress in cardiovascular diseases. Circ J 2009;73:411–8. 10.1253/circj.CJ-08-1102 19194043

[28] Sandner P, Becker-Pelster EM, Stasch JP. Discovery and development of sGC stimulators for the treatment of pulmonary hypertension and rare diseases. Nitric Oxide 2018;77:88–95. 10.1016/j.niox.2018.05.001 29738821

[29] Wobst J, Schunkert H, Kessler T. Genetic alterations in the NO-cGMP pathway and cardiovascular risk. Nitric Oxide 2018;76:105–12. 10.1016/j.niox.2018.03.019 29601927

[30] Erdmann J, Stark K, Esslinger UB, Rumpf PM, Koesling D, de Wit C, et al Dysfunctional nitric oxide signalling increases risk of myocardial infarction. Nature 2013;504:432–6. 10.1038/nature12722 24213632

[31] Emdin CA, Khera AV, Klarin D, Natarajan P, Zekavat SM, Nomura A, et al Phenotypic consequences of a genetic predisposition to enhanced nitric oxide signaling. Circulation 2018;137:222–32. 10.1161/circulationaha.117.028021 28982690 PMC5771958

[32] Farah C, Michel LYM, Balligand JL. Nitric oxide signalling in cardiovascular health and disease. Nat Rev Cardiol 2018;15:292–316. 10.1038/nrcardio.2017.224 29388567

[33] Mónica FZ, Bian K, Murad F. The endothelium-dependent nitric oxide-cGMP pathway. Adv Pharmacol 2016;77:1–27. 10.1016/bs.apha.2016.05.001 27451093

[34] Montfort WR, Wales JA, Weichsel A. Structure and activation of soluble guanylyl cyclase, the nitric oxide sensor. Antioxid Redox Signal 2017;26:107–21. 10.1089/ars.2016.6693 26979942 PMC5240008

[35] Sandner P, Follmann M, Becker-Pelster E, Hahn MG, Meier C, Freitas C, et al Soluble GC stimulators and activators: past, present and future. Br J Pharmacol 2021;181:4130–51. 10.1111/bph.15698 34600441

[36] European Medicines Agency . *Adempas Summary of Product Characteristics*. 2022. https://www.ema.europa.eu/en/documents/product-information/adempas-epar-product-information_en.pdf (31 July 2023, date last accessed).

[37] Akebia . *Akebia Announces Establishment of Rare Kidney Disease Pipeline*. 2025. https://ir.akebia.com/news-releases/news-release-details/akebia-announces-establishment-rare-kidney-disease-pipeline (13 January 2026, date last accessed).

[38] Mollace R, Scarano F, Bava I, Carresi C, Maiuolo J, Tavernese A, et al Modulation of the nitric oxide/cGMP pathway in cardiac contraction and relaxation: potential role in heart failure treatment. Pharmacological Research Epub 2023;196:106931. 10.1016/j.phrs.2023.106931 37722519

[39] Feil R, Lehners M, Stehle D, Feil S. Visualising and understanding cGMP signals in the cardiovascular system. Br J Pharmacol 2021;179:2394–412. doi:doi: 10.1111/bph.15500 33880767

[40] Numata G, Takimoto E. Cyclic GMP and PKG signaling in heart failure. Front Pharmacol 2022;13:792798. 10.3389/fphar.2022.792798 35479330 PMC9036358

[41] Blanton RM . cGMP signaling and modulation in heart failure. J Cardiovasc Pharmacol 2020;75:385–98. 10.1097/FJC.0000000000000749 31464774 PMC7044023

[42] Adler J, Kuret A, Längst N, Lukowski R. Targets of cGMP/cGKI in cardiac myocytes. J Cardiovasc Pharmacol 2020;75:494–507. 10.1097/fjc.0000000000000817 32168155

[43] Schlossmann J, Desch M. IRAG and novel PKG targeting in the cardiovascular system. Am J Physiol Heart Circ Physiol 2011;301:H672–82. 10.1152/ajpheart.00198.2011 21666108

[44] Pavlaki N, Nikolaev VO. Imaging of PDE2- and PDE3-mediated cGMP-to-cAMP cross-talk in cardiomyocytes. J Cardiovasc Dev Dis 2018;5:4. 10.3390/jcdd5010004 29367582 PMC5872352

[45] Kamel R, Leroy J, Vandecasteele G, Fischmeister R. Cyclic nucleotide phosphodiesterases as therapeutic targets in cardiac hypertrophy and heart failure. Nat Rev Cardiol 2023;20:90–108. 10.1038/s41569-022-00756-z 36050457

[46] Lukowski R, Feil R. Recent developments in cGMP research: from mechanisms to medicines and back. Br J Pharmacol 2022;179:2321–7. 10.1111/bph.15824 35332531

[47] Ahluwalia A, MacAllister RJ, Hobbs AJ. Vascular actions of natriuretic peptides. Cyclic GMP-dependent and -independent mechanisms. Basic Res Cardiol 2004;99:83–9. 10.1007/s00395-004-0459-6 14963666

[48] Buglioni A, Burnett JC Jr. New pharmacological strategies to increase cGMP. Annu Rev Med 2016;67:229–43. 10.1146/annurev-med-052914-091923 26473417

[49] Bork NI, Molina CE, Nikolaev VO. cGMP signalling in cardiomyocyte microdomains. Biochem Soc Trans 2019;47:1327–39. 10.1042/BST20190225 31652306

[50] Chen Y, Zheng Y, Iyer SR, Harders GE, Pan S, Chen HH, et al C53: a novel particulate guanylyl cyclase B receptor activator that has sustained activity in vivo with anti-fibrotic actions in human cardiac and renal fibroblasts. J Mol Cell Cardiol 2019;130:140–50. 10.1016/j.yjmcc.2019.03.024 30954448 PMC6557127

[51] Ichiki T, Burnett JC Jr. Atrial natriuretic peptide—old but new therapeutic in cardiovascular diseases. Circ J 2017;81:913–9. 10.1253/circj.CJ-17-0499 28552863

[52] European Medicines Agency . Entresto Summary of Product Characteristics. 2015. https://www.ema.europa.eu/en/documents/product-information/entresto-epar-product-information_en.pdf (10 April 2024, date last accessed).

[53] Food and Drug Administration . Entresto: Highlights of Prescribing Information. 2024. https://www.accessdata.fda.gov/drugsatfda_docs/label/2024/218591Orig1s000,207620Orig1s025lbl.pdf (19 June 2024, date last accessed).

[54] Chen Y, Burnett JC. Particulate guanylyl cyclase A/cGMP signaling pathway in the kidney: physiologic and therapeutic indications. Int J Mol Sci 2018;19:1006. 10.3390/ijms19041006 29584705 PMC5979439

[55] Schlossmann J, Schinner E. cGMP becomes a drug target. Naunyn Schmiedebergs Arch Pharmacol 2012;385:243–52. 10.1007/s00210-012-0730-6 22297800 PMC3281996

[56] Friebe A, Voussen B, Groneberg D. NO-GC in cells ‘off the beaten track’. Nitric Oxide 2018;77:12–8. 10.1016/j.niox.2018.03.020 29626542

[57] Frankenreiter S, Groneberg D, Kuret A, Krieg T, Ruth P, Friebe A, et al Cardioprotection by ischemic postconditioning and cyclic guanosine monophosphate-elevating agents involves cardiomyocyte nitric oxide-sensitive guanylyl cyclase. Cardiovasc Res 2018;114:822–9. 10.1093/cvr/cvy039 29438488

[58] Bork NI, Nikolaev VO. cGMP signaling in the cardiovascular system—the role of compartmentation and its live cell imaging. Int J Mol Sci 2018;19:801. 10.3390/ijms19030801 29534460 PMC5877662

[59] Menges L, Krawutschke C, Füchtbauer E, Füchtbauer A, Sandner P, Koesling D, et al Mind the gap (junction): cGMP induced by nitric oxide in cardiac myocytes originates from cardiac fibroblasts. Br J Pharmacol 2019;176:4696–707. 10.1111/bph.14835 31423565 PMC6965686

[60] Fukuma N, Tzimas C, Russo I, Dun W, Lance ML, Zhang Y, et al Cardiomyocyte GC1 mediates estrogenic angiogenesis in right heart remodeling. Circ Res 2025;137:1407–21. 10.1161/circresaha.124.326070 41127907 PMC13641253

[61] Breitenstein S, Roessig L, Sandner P, Lewis KS. Novel sGC stimulators and sGC activators for the treatment of heart failure. Handb Exp Pharmacol 2017;243:225–47. 10.1007/164_2016_100 27900610

[62] Cortese-Krott MM, Mergia E, Kramer CM, Luckstadt W, Yang J, Wolff G, et al Identification of a soluble guanylate cyclase in RBCs: preserved activity in patients with coronary artery disease. Redox Biol 2018;14:328–37. 10.1016/j.redox.2017.08.020 29024896 PMC5975213

[63] Jiao T, Collado A, Mahdi A, Tengbom J, Tratsiakovich Y, Milne GT, et al Stimulation of erythrocyte soluble guanylyl cyclase induces cGMP export and cardioprotection in type 2 diabetes. JACC Basic Transl Sci 2023;8:907–18. 10.1016/j.jacbts.2023.02.017 37719424 PMC10504399

[64] Ahluwalia A, Foster P, Scotland RS, McLean PG, Mathur A, Perretti M, et al Antiinflammatory activity of soluble guanylate cyclase: cGMP-dependent down-regulation of P-selectin expression and leukocyte recruitment. Proc Natl Acad Sci U S A 2004;101:1386–91. 10.1073/pnas.0304264101 14742866 PMC337062

[65] Vandenwijngaert S, Swinnen M, Walravens AS, Beerens M, Gillijns H, Caluwé E, et al Decreased soluble guanylate cyclase contributes to cardiac dysfunction induced by chronic doxorubicin treatment in mice. Antioxid Redox Signal 2017;26:153–64. 10.1089/ars.2015.6542 27505125 PMC5278809

[66] Masuyama H, Tsuruda T, Sekita Y, Hatakeyama K, Imamura T, Kato J, et al Pressure-independent effects of pharmacological stimulation of soluble guanylate cyclase on fibrosis in pressure-overloaded rat heart. Hypertens Res 2009;32:597–603. 10.1038/hr.2009.64 19424280

[67] Beyer C, Zenzmaier C, Palumbo-Zerr K, Mancuso R, Distler A, Dees C, et al Stimulation of the soluble guanylate cyclase (sGC) inhibits fibrosis by blocking non-canonical TGFbeta signalling. Ann Rheum Dis 2015;74:1408–16. 10.1136/annrheumdis-2013-204508 24567525

[68] Friebe A, Sandner P, Schmidtko A. cGMP: a unique 2nd messenger molecule—recent developments in cGMP research and development. Naunyn Schmiedebergs Arch Pharmacol 2020;393:287–302. 10.1007/s00210-019-01779-z 31853617 PMC7260148

[69] Buys ES, Sips P, Vermeersch P, Raher MJ, Rogge E, Ichinose F, et al Gender-specific hypertension and responsiveness to nitric oxide in sGCalpha1 knockout mice. Cardiovasc Res 2008;79:179–86. 10.1093/cvr/cvn068 18339647

[70] Cawley SM, Kolodziej S, Ichinose F, Brouckaert P, Buys ES, Bloch KD. sGCα_1_ mediates the negative inotropic effects of NO in cardiac myocytes independent of changes in calcium handling. Am J Physiol Heart Circ Physiol 2011;301:H157–63. 10.1152/ajpheart.01273.2010 21536853 PMC3129918

[71] Vettel C, Lämmle S, Ewens S, Cervirgen C, Emons J, Ongherth A, et al PDE2-mediated cAMP hydrolysis accelerates cardiac fibroblast to myofibroblast conversion and is antagonized by exogenous activation of cGMP signaling pathways. Am J Physiol Heart Circ Physiol 2014;306:H1246–52. 10.1152/ajpheart.00852.2013 24531807

[72] Krüger M, Kötter S, Grützner A, Lang P, Andresen C, Redfield MM, et al Protein kinase G modulates human myocardial passive stiffness by phosphorylation of the titin springs. Circ Res 2009;104:87–94. 10.1161/circresaha.108.184408 19023132

[73] Borbély A, Falcao-Pires I, van Heerebeek L, Hamdani N, Edes I, Gavina C, et al Hypophosphorylation of the Stiff N2B titin isoform raises cardiomyocyte resting tension in failing human myocardium. Circ Res 2009;104:780–6. 10.1161/circresaha.108.193326 19179657

[74] Fan D, Takawale A, Lee J, Kassiri Z. Cardiac fibroblasts, fibrosis and extracellular matrix remodeling in heart disease. Fibrogenesis Tissue Repair 2012;5:15. 10.1186/1755-1536-5-15 22943504 PMC3464725

[75] van Heerebeek L, Hamdani N, Falcão-Pires I, Leite-Moreira AF, Begieneman MP, Bronzwaer JG, et al Low myocardial protein kinase G activity in heart failure with preserved ejection fraction. Circulation 2012;126:830–9. 10.1161/circulationaha.111.076075 22806632

[76] Lukowski R, Krieg T, Rybalkin SD, Beavo J, Hofmann F. Turning on cGMP-dependent pathways to treat cardiac dysfunctions: boom, bust, and beyond. Trends Pharmacol Sci 2014;35:404–13. 10.1016/j.tips.2014.05.003 24948380

[77] Schwaerzer GK, Casteel DE, Cividini F, Kalyanaraman H, Zhuang S, Gu Y, et al Constitutive protein kinase G activation exacerbates stress-induced cardiomyopathy. Br J Pharmacol 2022;179:2413–29. 10.1111/bph.15530 34000062 PMC9926932

[78] Chan MH, Aminzai S, Hu T, Taran A, Li S, Kim C, et al A substitution in cGMP-dependent protein kinase 1 associated with aortic disease induces an active conformation in the absence of cGMP. J Biol Chem 2020;295:10394–405. 10.1074/jbc.RA119.010984 32506052 PMC7383375

[79] Guo DC, Regalado E, Casteel DE, Santos-Cortez RL, Gong L, Kim JJ, et al Recurrent gain-of-function mutation in PRKG1 causes thoracic aortic aneurysms and acute aortic dissections. Am J Hum Genet 2013;93:398–404. 10.1016/j.ajhg.2013.06.019 23910461 PMC3738837

[80] Frantz S, Klaiber M, Baba HA, Oberwinkler H, Volker K, Gabetaner B, et al Stress-dependent dilated cardiomyopathy in mice with cardiomyocyte-restricted inactivation of cyclic GMP-dependent protein kinase I. Eur Heart J 2013;34:1233–44. 10.1093/eurheartj/ehr445 22199120 PMC3631523

[81] Patrucco E, Domes K, Sbroggio M, Blaich A, Schlossmann J, Desch M, et al Roles of cGMP-dependent protein kinase I (cGKI) and PDE5 in the regulation of Ang II-induced cardiac hypertrophy and fibrosis. Proc Natl Acad Sci U S A 2014;111:12925–9. 10.1073/pnas.1414364111 25139994 PMC4156763

[82] Santos MC, Birkenfeld L, Pham T, Maier S, Paulus K, Ullemeyer L, et al Angiotensin II-induced cardiac fibrosis and dysfunction are exacerbated by deletion of cGKI in periostin+ myofibroblasts. Clin Sci (Lond) 2025;139:507–26. 10.1042/cs20241204 40267335 PMC12247847

[83] Calamaras TD, Pande S, Baumgartner RA, Kim SK, McCarthy JC, Martin GL, et al MLK3 mediates impact of PKG1α on cardiac function and controls blood pressure through separate mechanisms. JCI Insight 2021;6:e149075. 10.1172/jci.insight.149075 34324442 PMC8492323

[84] Melichar VO, Behr-Roussel D, Zabel U, Uttenthal LO, Rodrigo J, Rupin A, et al Reduced cGMP signaling associated with neointimal proliferation and vascular dysfunction in late-stage atherosclerosis. Proc Natl Acad Sci U S A 2004;101:16671–6. 10.1073/pnas.0405509101 15546990 PMC534521

[85] Zhang YH, Jin CZ, Jang JH, Wang Y. Molecular mechanisms of neuronal nitric oxide synthase in cardiac function and pathophysiology. J Physiol 2014;592:3189–200. 10.1113/jphysiol.2013.270306 24756636 PMC4146369

[86] Denham NC, Pearman CM, Caldwell JL, Madders GWP, Eisner DA, Trafford AW, et al Calcium in the pathophysiology of atrial fibrillation and heart failure. Front Physiol 2018;9:1380. 10.3389/fphys.2018.01380 30337881 PMC6180171

[87] Voigt N, Heijman J, Wang Q, Chiang DY, Li N, Karck M, et al Cellular and molecular mechanisms of atrial arrhythmogenesis in patients with paroxysmal atrial fibrillation. Circulation 2014;129:145–56. 10.1161/circulationaha.113.006641 24249718 PMC4342412

[88] Voigt N, Li N, Wang Q, Wang W, Trafford AW, Abu-Taha I, et al Enhanced sarcoplasmic reticulum Ca2+ leak and increased Na+-Ca2+ exchanger function underlie delayed afterdepolarizations in patients with chronic atrial fibrillation. Circulation 2012;125:2059–70. 10.1161/circulationaha.111.067306 22456474 PMC4663993

[89] Li N, Chiang DY, Wang S, Wang Q, Sun L, Voigt N, et al Ryanodine receptor-mediated calcium leak drives progressive development of an atrial fibrillation substrate in a transgenic mouse model. Circulation 2014;129:1276–85. 10.1161/circulationaha.113.006611 24398018 PMC4026172

[90] Chelu MG, Sarma S, Sood S, Wang S, van Oort RJ, Skapura DG, et al Calmodulin kinase II-mediated sarcoplasmic reticulum Ca2+ leak promotes atrial fibrillation in mice. J Clin Invest 2009;119:1940–51. 10.1172/jci37059 19603549 PMC2701862

[91] Münzel T, Daiber A, Gori T. Nitrate therapy: new aspects concerning molecular action and tolerance. Circulation 2011;123:2132–44. 10.1161/circulationaha.110.981407 21576678

[92] Stasch JP, Hobbs AJ. NO-independent, haem-dependent soluble guanylate cyclase stimulators. Handb Exp Pharmacol 2009;191:277–308. 10.1007/978-3-540-68964-5_13 19089334

[93] Ignarro LJ, Napoli C, Loscalzo J. Nitric oxide donors and cardiovascular agents odulating the bioactivity of nitric oxide. Circ Res 2002;90:21–8. doi:10.1161/hh0102.102330 11786514

[94] Remington J, Winters K. Effectiveness of dietary inorganic nitrate for lowering blood pressure in hypertensive adults: a systematic review. JBI Database System Rev Implement Rep 2019;17:365–89. 10.11124/JBISRIR-2017-003842 30870330

[95] Hopper I, Connell C, Briffa T, De Pasquale CG, Driscoll A, Kistler PM, et al Nutraceuticals in patients with heart failure: a systematic review. J Card Fail 2020;26:166–79. 10.1016/j.cardfail.2019.10.014 31704198

[96] European Medicines Agency . Viagra Summary of Product Characteristics. 2023. https://www.ema.europa.eu/en/documents/product-information/viagra-epar-product-information_en.pdf (31 July 2023, date last accessed).

[97] European Medicines Agency . Levitra Summary of Product Characteristics. 2021. https://www.ema.europa.eu/en/documents/product-information/levitra-epar-product-information_en.pdf (18 August 2023, date last accessed).

[98] European Medicines Agency . Cialis Summary of Product Characteristics. 2021. https://www.ema.europa.eu/en/documents/product-information/cialis-epar-product-information_en.pdf (18 August 2023, date last accessed).

[99] U.S. Food and Drug Administration . Drug Approval Package: Cialis (tadalafil) Tablets. 2004. https://www.accessdata.fda.gov/drugsatfda_docs/label/2018/021368s030lbl.pdf (30 January 2026, date last accessed).

[100] U.S. Food and Drug Administration . *Drug Approval Package: Levitra (vardenafil) Tablets*. 2003. https://www.accessdata.fda.gov/drugsatfda_docs/label/2023/021400s023lbl.pdf (30 January 2026, date last accessed).

[101] European Medicines Agency . Revatio Summary of Product Characteristics. 2023. https://www.ema.europa.eu/en/documents/product-information/revatio-epar-product-information_en.pdf (31 July 2023, date last accessed).

[102] European Medicines Agency . Adcirca Summary of Product Characteristics. 2008. https://www.ema.europa.eu/en/documents/product-information/adcirca-epar-product-information_en.pdf (10 April 2008, date last accessed).

[103] Shabsigh R . Therapy of ED: PDE-5 inhibitors. Endocrine 2004;23:135–41. 10.1385/endo:23:2-3:135 15146092

[104] Bruzziches R, Francomano D, Gareri P, Lenzi A, Aversa A. An update on pharmacological treatment of erectile dysfunction with phosphodiesterase type 5 inhibitors. Expert Opin Pharmacother 2013;14:1333–44. 10.1517/14656566.2013.799665 23675780

[105] Oudiz R, Shapiro S, Torres F, Feldman J, Frost A, Allard M, et al ATHENA-1: hemodynamic improvements following the addition of ambrisentan to background PDE5i therapy in patients with pulmonary arterial hypertension. CHEST 2011;140:905A. 10.1378/chest.1113577 28427554

[106] Hoeper MM, Simonneau G, Corris PA, Ghofrani HA, Klinger JR, Langleben D, et al RESPITE: switching to riociguat in pulmonary arterial hypertension patients with inadequate response to phosphodiesterase-5 inhibitors. Eur Respir J 2017;50:1602425. 10.1183/13993003.02425-2016 28889107 PMC5898946

[107] Shapiro S, Torres F, Feldman J, Keogh A, Allard M, Blair C, et al Clinical and hemodynamic improvements after adding ambrisentan to background PDE5i therapy in patients with pulmonary arterial hypertension exhibiting a suboptimal therapeutic response (ATHENA-1). Respir Med 2017;126:84–92. 10.1016/j.rmed.2017.03.025 28427554

[108] Hoeper MM, Klinger JR, Benza RL, Simonneau G, Langleben D, Naeije R, et al Rationale and study design of RESPITE: an open-label, phase 3b study of riociguat in patients with pulmonary arterial hypertension who demonstrate an insufficient response to treatment with phosphodiesterase-5 inhibitors. Respir Med 2017;122:S18–22. 10.1016/j.rmed.2016.11.001 27887774

[109] Garban H, Vernet D, Freedman A, Rajfer J, Gonzalez-Cadavid N. Effect of aging on nitric oxide-mediated penile erection in rats. Am J Physiol 1995;268:H467–75. 10.1152/ajpheart.1995.268.1.H467 7530924

[110] Mulhall J, Teloken P, Brock G, Kim E. Obesity, dyslipidemias and erectile dysfunction: a report of a subcommittee of the sexual medicine society of North America. J Sex Med 2006;3:778–86. 10.1111/j.1743-6109.2006.00286.x 16942522

[111] Gurbuz N, Mammadov E, Usta MF. Hypogonadism and erectile dysfunction: an overview. Asian J Androl 2008;10:36–43. 10.1111/j.1745-7262.2008.00375.x 18087642

[112] Cartledge JJ, Eardley I, Morrison JF. Nitric oxide-mediated corpus cavernosal smooth muscle relaxation is impaired in ageing and diabetes. BJU Int 2001;87:394–401. 10.1046/j.1464-410x.2001.00065.x 11251538

[113] Musicki B, Burnett AL. Endothelial dysfunction in diabetic erectile dysfunction. Int J Impot Res 2007;19:129–38. 10.1038/sj.ijir.3901494 16775612

[114] Takimoto E, Champion HC, Li M, Belardi D, Ren S, Rodriguez ER, et al Chronic inhibition of cyclic GMP phosphodiesterase 5A prevents and reverses cardiac hypertrophy. Nat Med 2005;11:214–22. 10.1038/nm1175 15665834

[115] Cooper TJ, Cleland JGF, Guazzi M, Pellicori P, Ben Gal T, Amir O, et al Effects of sildenafil on symptoms and exercise capacity for heart failure with reduced ejection fraction and pulmonary hypertension (the SilHF study): a randomized placebo-controlled multicentre trial. Eur J Heart Fail 2022;24:1239–48. 10.1002/ejhf.2527 35596935 PMC9544113

[116] Hoendermis ES, Liu LC, Hummel YM, van der Meer P, de Boer RA, Berger RM, et al Effects of sildenafil on invasive haemodynamics and exercise capacity in heart failure patients with preserved ejection fraction and pulmonary hypertension: a randomized controlled trial. Eur Heart J 2015;36:2565–73. 10.1093/eurheartj/ehv336 26188003

[117] Wunder F, Tersteegen A, Rebmann A, Erb C, Fahrig T, Hendrix M. Characterization of the first potent and selective PDE9 inhibitor using a cGMP reporter cell line. Mol Pharmacol 2005;68:1775–81. 10.1124/mol.105.017608 16150925

[118] Meibom D, Micus S, Andreevski AL, Anlauf S, Bogner P, von Buehler CJ, et al BAY-7081: a potent, selective, and orally bioavailable cyanopyridone-based PDE9A inhibitor. J Med Chem 2022;65:16420–31. 10.1021/acs.jmedchem.2c01267 36475653 PMC9791655

[119] Lee DI, Zhu G, Sasaki T, Cho GS, Hamdani N, Holewinski R, et al Phosphodiesterase 9A controls nitric-oxide-independent cGMP and hypertrophic heart disease. Nature 2015;519:472–6. 10.1038/nature14332 25799991 PMC4376609

[120] Ko FN, Wu CC, Kuo SC, Lee FY, Teng CM. YC-1, a novel activator of platelet guanylate cyclase. Blood 1994;84:4226–33. 10.1182/blood.V84.12.4226.bloodjournal84124226 7527671

[121] Mülsch A, Bauersachs J, Schäfer A, Stasch JP, Kast R, Busse R. Effect of YC-1, an NO-independent, superoxide-sensitive stimulator of soluble guanylyl cyclase, on smooth muscle responsiveness to nitrovasodilators. Br J Pharmacol 1997;120:681–9. 10.1038/sj.bjp.0700982 9051308 PMC1564520

[122] Follmann M, Ackerstaff J, Redlich G, Wunder F, Lang D, Kern A, et al Discovery of the soluble guanylate cyclase stimulator vericiguat (BAY 1021189) for the treatment of chronic heart failure. J Med Chem 2017;60:5146–61. 10.1021/acs.jmedchem.7b00449 28557445

[123] Khaybullina D, Patel A, Zerilli T. Riociguat (adempas): a novel agent for the treatment of pulmonary arterial hypertension and chronic thromboembolic pulmonary hypertension. P T 2014;39:749–58. 25395817 PMC4218670

[124] Khan MS, Shahid I, Greene SJ, Mentz RJ, DeVore AD, Butler J. Mechanisms of current therapeutic strategies for heart failure: more questions than answers? Cardiovasc Res 2023;118:3467–81. 10.1093/cvr/cvac187 36536991

[125] Greene SJ, Bauersachs J, Brugts JJ, Ezekowitz JA, Filippatos G, Gustafsson F, et al Management of worsening heart failure with reduced ejection fraction: JACC focus seminar 3/3. J Am Coll Cardiol 2023;82:559–71. 10.1016/j.jacc.2023.04.057 37532426

[126] Cannan CR, McGoon MD, Holmes DR Jr, Lerman A. Altered coronary endothelial function in a patient with asymptomatic left ventricular dysfunction. Int J Cardiol 1996;53:147–51. 10.1016/0167-5273(95)02513-8 8682600

[127] Bellien J, Favre J, Iacob M, Gao J, Thuillez C, Richard V, et al Arterial stiffness is regulated by nitric oxide and endothelium-derived hyperpolarizing factor during changes in blood flow in humans. Hypertension 2010;55:674–80. 10.1161/hypertensionaha.109.142190 20083732

[128] Zeiher AM, Drexler H, Wollschlaeger H, Saurbier B, Just H. Coronary vasomotion in response to sympathetic stimulation in humans: importance of the functional integrity of the endothelium. J Am Coll Cardiol 1989;14:1181–90. 10.1016/0735-1097(89)90414-2 2808971

[129] Nägele MP, Barthelmes J, Ludovici V, Cantatore S, von Eckardstein A, Enseleit F, et al Retinal microvascular dysfunction in heart failure. Eur Heart J 2018;39:47–56. 10.1093/eurheartj/ehx565 29069316

[130] Barthelmes J, Nägele MP, Cantatore S, Novruzov E, Ludovici V, von Eckardstein A, et al Retinal microvascular dysfunction in patients with coronary artery disease with and without heart failure: a continuum? Eur J Heart Fail 2019;21:988–97. 10.1002/ejhf.1537 31271256

[131] Follmann M, Becker C, Rossig L, Sandner P, Stasch JP. Discovery and development of the soluble guanylate cyclase stimulator vericiguat for the treatment of chronic heart failure (eds.), *Contemporary Accounts in Drug Discovery and Development*. Hoboken, NJ, USA: Wiley and Sons, 2021, 27–50.

[132] LoBue A, Heuser SK, Lindemann M, Li J, Rahman M, Kelm M, et al Red blood cell endothelial nitric oxide synthase: a major player in regulating cardiovascular health. Br J Pharmacol 2026;183:40–56. 10.1111/bph.16230 37658519

[133] Yang J, Sundqvist ML, Zheng X, Jiao T, Collado A, Tratsiakovich Y, et al Hypoxic erythrocytes mediate cardioprotection through activation of soluble guanylate cyclase and release of cyclic GMP. J Clin Invest 2023;133:e167693. 10.1172/JCI167693 37655658 PMC10471167

[134] Gladwin MT, Gordeuk VR, Desai PC, Minniti C, Novelli EM, Morris CR, et al Riociguat in patients with sickle cell disease and hypertension or proteinuria (STERIO-SCD): a randomised, double-blind, placebo controlled, phase 1-2 trial. Lancet Haematol 2024;11:e345–57. 10.1016/S2352-3026(24)00045-0 38554715 PMC12053730

[135] Irvine JC, Ganthavee V, Love JE, Alexander AE, Horowitz JD, Stasch JP, et al The soluble guanylyl cyclase activator Bay 58-2667 selectively limits cardiomyocyte hypertrophy. PLoS One 2012;7:e44481. 10.1371/journal.pone.0044481 23144773 PMC3492396

[136] Masuyama H, Tsuruda T, Kato J, Imamura T, Asada Y, Stasch JP, et al Soluble guanylate cyclase stimulation on cardiovascular remodeling in angiotensin II-induced hypertensive rats. Hypertension 2006;48:972–8. 10.1161/01.Hyp.0000241087.12492.47 16982964

[137] Rüdebusch J, Benkner A, Nath N, Fleuch L, Kaderali L, Grube K, et al Stimulation of soluble guanylyl cyclase (sGC) by riociguat attenuates heart failure and pathological cardiac remodelling. Br J Pharmacol 2020;179:2430–42. 10.1111/bph.15333 33247945

[138] Mathar I, Pavkovic M, Scheerer N, Hartmann E, Sandner P. The sGC stimulator vericiguat improved outcome in a rodent model of heart failure with preserved ejection fraction (HFpEF). Circulation 2018;138:A15553.

[139] Lukowski R, Rybalkin SD, Loga F, Leiss V, Beavo JA, Hofmann F. Cardiac hypertrophy is not amplified by deletion of cGMP-dependent protein kinase I in cardiomyocytes. Proc Natl Acad Sci U S A 2010;107:5646–51. 10.1073/pnas.1001360107 20212138 PMC2851748

[140] Menges L, Giesen J, Yilmaz K, Mergia E, Fuchtbauer A, Fuchtbauer EM, et al It takes two to tango: cardiac fibroblast-derived NO-induced cGMP enters cardiac myocytes and increases cAMP by inhibiting PDE3. Commun Biol 2023;6:504. 10.1038/s42003-023-04880-5 37165086 PMC10172304

[141] Costell MH, Ancellin N, Bernard RE, Zhao S, Upson JJ, Morgan LA, et al Comparison of soluble guanylate cyclase stimulators and activators in models of cardiovascular disease associated with oxidative stress. Front Pharmacol 2012;3:128. 10.3389/fphar.2012.00128 22783192 PMC3389674

[142] Lou Q, Li L, Liu G, Li T, Zhang L, Zang Y, et al Vericiguat reduces electrical and structural remodeling in a rabbit model of atrial fibrillation. J Cardiovasc Pharmacol Ther 2023;28:10742484231185252. 10.1177/10742484231185252 37403470

[143] Tobin JV, Zimmer DP, Shea C, Germano P, Bernier SG, Liu G, et al Pharmacological characterization of IW-1973, a novel soluble guanylate cyclase stimulator with extensive tissue distribution, antihypertensive, anti-inflammatory, and antifibrotic effects in preclinical models of disease. J Pharmacol Exp Ther 2018;365:664–75. 10.1124/jpet.117.247429 29643251

[144] Liu G, Shea CM, Jones JE, Price GM, Warren W, Lonie E, et al Praliciguat inhibits progression of diabetic nephropathy in ZSF1 rats and suppresses inflammation and apoptosis in human renal proximal tubular cells. Am J Physiol Renal Physiol 2020;319:F697–711. 10.1152/ajprenal.00003.2020 32865013

[145] European Medicines Agency . *Verquvo^TM^ Summary of Opinion (Initial Authorisation)*. 2021. https://www.ema.europa.eu/en/documents/smop-initial/chmp-summary-positive-opinion-verquvo_en.pdf (01 December 2022, date last accessed).

[146] Gheorghiade M, Greene SJ, Butler J, Filippatos G, Lam CS, Maggioni AP, et al Effect of vericiguat, a soluble guanylate cyclase stimulator, on natriuretic peptide levels in patients with worsening chronic heart failure and reduced ejection fraction: the SOCRATES-REDUCED randomized trial. JAMA 2015;314:2251–62. 10.1001/jama.2015.15734 26547357

[147] Pieske B, Butler J, Filippatos G, Lam C, Maggioni PA, Ponikowski P, et al Rationale and design of the SOluble guanylate Cyclase stimulatoR in heArT failurE Studies (SOCRATES). Eur J Heart Fail 2014;16:1026–38. 10.1002/ejhf.135 25056511

[148] Armstrong PW, Pieske B, Anstrom KJ, Ezekowitz J, Hernandez AF, Butler J, et al Vericiguat in patients with heart failure and reduced ejection fraction. N Engl J Med 2020;382:1883–93. 10.1056/NEJMoa1915928 32222134

[149] Armstrong PW, Roessig L, Patel MJ, Anstrom KJ, Butler J, Voors AA, et al A multicenter, randomized, double-blind, placebo-controlled trial of the efficacy and safety of the oral soluble guanylate cyclase stimulator: the VICTORIA trial. JACC Heart Fail 2018;6:96–104. 10.1016/j.jchf.2017.08.013 29032136

[150] Butler J, McMullan CJ, Anstrom KJ, Barash I, Bonaca MP, Borentain M, et al Vericiguat in patients with chronic heart failure and reduced ejection fraction (VICTOR): a double-blind, placebo-controlled, randomised, phase 3 trial. Lancet 2025;406:1341–50. 10.1016/S0140-6736(25)01665-4 40897189

[151] Ezekowitz JA, O'Connor CM, Troughton RW, Alemayehu WG, Westerhout CM, Voors AA, et al N-terminal pro-B-type natriuretic peptide and clinical outcomes: vericiguat heart failure with reduced ejection fraction study. JACC Heart Fail 2020;8:931–9. 10.1016/j.jchf.2020.08.008 33039447

[152] Lam CSP, Pina IL, Zheng Y, Bonderman D, Pouleur AC, Saldarriaga C, et al Age, sex, and outcomes in heart failure with reduced EF: insights from the VICTORIA trial. JACC Heart Fail 2023;11:1246–57. 10.1016/j.jchf.2023.06.020 37565973

[153] Butler J, Zheng Y, Khan MS, Bonderman D, Lund LH, deFilippi CR, et al Ejection fraction, biomarkers, and outcomes and impact of vericiguat on outcomes across EF in VICTORIA. JACC Heart Fail 2023;11:583–92. 10.1016/j.jchf.2022.12.014 37137660

[154] Saldarriaga C, Atar D, Stebbins A, Lewis BS, Abidin IZ, Blaustein RO, et al Vericiguat in patients with coronary artery disease and heart failure with reduced ejection fraction. Eur J Heart Fail 2022;24:782–90. 10.1002/ejhf.2468 35239245

[155] Lam CSP, Mulder H, Lopatin Y, Vazquez-Tanus JB, Siu D, Ezekowitz J, et al Blood pressure and safety events with vericiguat in the VICTORIA trial. J Am Heart Assoc 2021;10:e021094. 10.1161/jaha.121.021094 34743540 PMC8751950

[156] Voors AA, Mulder H, Reyes E, Cowie MR, Lassus J, Hernandez AF, et al Renal function and the effects of vericiguat in patients with worsening heart failure with reduced ejection fraction: insights from the VICTORIA (Vericiguat Global Study in Subjects with HFrEF) trial. Eur J Heart Fail 2021;23:1313–21. 10.1002/ejhf.2221 33999486 PMC8453520

[157] Ezekowitz JA, McMullan CJ, Westerhout CM, Pina IL, Lopez-Sendon J, Anstrom KJ, et al Background medical therapy and clinical outcomes from the VICTORIA trial. Circ Heart Fail 2023;16:e010599. 10.1161/CIRCHEARTFAILURE.123.010599 37417824

[158] Ezekowitz J, Alemayehu W, Edelmann F, Ponikowski P, Lam CSP, O'Connor CM, et al Diuretic use and outcomes in patients with heart failure with reduced ejection fraction: insights from the VICTORIA trial. Eur J Heart Fail 2024;26:628–37. 10.1002/ejhf.3179 38450878

[159] Senni M, Alemayehu WG, Sim D, Edelmann F, Butler J, Ezekowitz J, et al Efficacy and safety of vericiguat in patients with heart failure with reduced ejection fraction treated with sacubitril/valsartan: insights from the VICTORIA trial. Eur J Heart Fail 2022;24:1614–22. 10.1002/ejhf.2608 35791083

[160] Böttcher M, Dungen HD, Corcea V, Donath F, Fuhr R, Gal P, et al Vericiguat: a randomized, phase ib, placebo-controlled, double-blind, QTc interval study in patients with chronic coronary syndromes. Am J Cardiovasc Drugs 2023;23:145–55. 10.1007/s40256-022-00557-2 36633816 PMC10006255

[161] Butler J, Fioretti F, McMullan CJ, Anstrom KJ, Barash I, Bonaca MP, et al Vericiguat and mortality in heart failure and reduced ejection fraction: the VICTOR trial. Eur Heart J 2025;47:683–97. 10.1093/eurheartj/ehaf655 PMC13089535 40884032

[162] Zannad F, O'Connor CM, Butler J, McMullan CJ, Anstrom KJ, Barash I, et al Vericiguat for patients with heart failure and reduced ejection fraction across the risk spectrum: an individual participant data analysis of the VICTORIA and VICTOR trials. Lancet 2025;406:1351–62. 10.1016/S0140-6736(25)01682-4 40897188

[163] Saldarriaga CI, Zannad F, McMullan CJ, Xing A, Gates D, Anstrom KJ, et al Baseline characteristics of contemporary trial participants with heart failure and reduced ejection fraction: the VICTOR trial. Eur J Heart Fail 2025;27:1426–35. 10.1002/ejhf.3598 39956649 PMC12482839

[164] Greene SJ, Fonarow GC, Butler J. Risk profiles in heart failure: baseline, residual, worsening, and advanced heart failure risk. Circ Heart Fail 2020;13:e007132. 10.1161/circheartfailure.120.007132 32482088

[165] Sandner P, Zimmer DP, Milne GT, Follmann M, Hobbs A, Stasch JP. Soluble guanylate cyclase stimulators and activators. Handb Exp Pharmacol 2021;264:355–94. 10.1007/164_2018_197 30689085

