# Supplementary material for: Use of soluble guanylyl cyclase stimulators in heart failure therapy—a mode of action perspective
Source: ESC Heart Fail. 2026 Apr 27;13(3):xvag117. doi: 10.1093/eschf/xvag117 (PMC13173434; doi:10.1093/eschf/xvag117)
Supplement: xvag117_Supplementary_Data [file xvag117_supplementary_data.docx]

**SUPPLEMENTARY MATERIALS**

**Part 1. Cellular responses and pathomechanisms targeted by soluble guanylyl cyclase (sGC) stimulators and the resulting local and systemic effects – preclinical data**

## Vascular smooth muscle cells (VSMCs), vasodilation, afterload and vascular stiffness

In isolated blood vessels of different species and of different vascular beds sGC stimulators concentration-dependently reduced vascular resistance,^1^ resulting in an afterload decrease. sGC stimulators could also increase local organ and tissue perfusion as shown in isolated hearts and kidneys.^1,2^

In a preclinical model of hypertension, vericiguat decreased aortic wave reflection parameters and augmentation index at doses that do not reduce blood pressure, suggesting an effect on muscular arteries.^3^

## VSMC effects versus inotropy

The increased levels of cyclic guanosine monophosphate (cGMP) induced by clinically relevant concentrations of riociguat had no direct effect on contraction or relaxation of cardiac myocytes from healthy rats *in vitro.*^4^

In *ex vivo* studies in isolated rat hearts, vericiguat increased coronary flow but had no effect on heart rate, contractility (dp/dt) or left ventricular (LV) diastolic pressure.^1^

## Smooth muscle cells/muscle cells and oxygen saturation

Impaired vasodilation reduces muscle perfusion and oxygen uptake in heart failure (HF). Indirect evidence of the benefits of sGC stimulators on skeletal muscle has come from preclinical studies in rodent models of Duchenne muscular dystrophy.^5^ but also from studies of sGC activators in HF models.^6^ In rats with HF with reduced ejection fraction (HFrEF), sGC activator administration improved blood flow in skeletal muscle by increasing capillary red blood cell flux and skeletal muscle O_2_ delivery via activation of oxidized sGC.^6^ Further studies are needed to evaluate the effect of sGC stimulators in skeletal myocytes.

## ROS production, cell adhesion and inflammation

Studies of the effects of increased cGMP on inflammatory cells are scarce. It has been shown early on that the anti-inflammatory effects of cGMP could be due to the downregulation of P-selectin, attenuating leucocyte recruitment.^7^ In addition, sGC stimulation reduced cytokine-induced leukocyte recruitment in mice^8^ and vericiguat attenuated reactive oxygen species (ROS) production in a rat model of mitral regurgitation.^9^ Interestingly, in clinical studies vericiguat also reduced C-reactive protein levels.^10,11^ However, future studies are required to investigate these potential anti-inflammatory effects and whether these effects are driven by potential interactions with the immune system and/or due to systemic improvements, for example, on vascular and cardiac function and perfusion.

## Cell adhesion and atherosclerosis

Loss of sGC function in platelets increased leukocyte recruitment and formation of atherosclerotic plaque in an atherosclerosis-prone mouse model.^12^ *In vitro* studies demonstrated that the increase of leukocyte adhesion to endothelial cells is mediated by a reduction in release of angiopoietin-1 by activated platelets lacking sGC.^12^ sGC stimulation with BAY-747 was shown to increase angiopoietin-1 release *in vitro* and reduce leukocyte adhesion and plaque formation in the atherosclerosis-prone mouse model.^12^ Platelet-derived growth factor signalling reduces sGC expression in VSMCs. This plays a key role in the migration of human VSMCs from the media to the intima of the vascular wall, the initial step in neointimal hyperplasia after vascular injury and in the progression of atherosclerosis.^13^

Pro-atherosclerotic effects of nitric oxide (NO)–sGC–cGMP signalling have also been reported. In atherosclerotic mice fed a high-fat diet, complete deficiency of *GUCY1A3*, which encodes the sGC alpha 1 (sGC-α1) subunit, was associated with a reduction in size and collagen content of atherosclerotic plaque.^14^ *In vitro* studies demonstrated that *GUCY1A3* deficiency prevented the phenotypic switching of aortic SMCs from a contractile to a pro-atherosclerotic synthetic state.^14^ Coding variants in *GUCY1A3* have been shown to lead to a reduction in cGMP that can be reversed by the sGC stimulator, BAY41-2272 *in vitro*.^15^

## Platelets and coagulation

Preclinical studies point towards the role of sGC in platelets in cardioprotection through reduced platelet activation and inflammation.^16^ Antiplatelet activity is well known to be associated with an increase in cAMP. Antiplatelet effects of riociguat have been demonstrated at supra-therapeutic doses as shown by experiments in murine washed platelets versus whole blood.^17^

## Adipocytes and adipose tissue differentiation

NO–sGC–cGMP signalling mediates brown-adipose tissue thermogenesis and promotes ‘browning’ of white adipose tissue,^18,19^ and is impaired in visceral adipose tissue of obese individuals.^20^ PKGI is a key receptor for mediating the effects of cGMP in brown-adipose tissue, and modifies the ability of NO and cGMP to induce mitochondrial biogenesis.^21^ PKGI also controls insulin signalling in brown-adipose tissue by inhibiting the activity of RhoA and Rho-associated kinases.^21^ *In vitro* and *in vivo* studies have demonstrated that vasodilator-stimulated phosphoprotein (VASP) deficiency is associated with increased activation of Rac1, increased sGC and cGMP concentrations, and enhanced brown-adipocyte differentiation and energy expenditure.^22^ A VASP–Rac–sGC feedback loop limits cGMP production, thereby regulating adipogenesis and energy homeostasis.^22^

The sGC stimulator BAY41-8543 increased lipid uptake and activity of murine brown adipose tissue, and stimulated ‘browning’ of murine white adipose tissue and human adipocytes.^18^ It protected against diet-induced weight gain in mice fed a high-fat diet, and induced weight loss in obese mice.^18^

## Glycaemic and metabolic control

cGMP-dependent PKGI (cGKI) has been identified as an endogenous modulator of glucose-induced inhibition glucagon release^23^ demonstrating a role of NO–sGC–cGMP signalling in glycaemic control. Gene-targeted mice that lack cGKI in pancreatic islet cells demonstrate abnormal glucagon secretion that is reflected in a hyperglycaemic fasting phenotype.^23^ *In vitro* studies suggest that NO/cGMP can increase glucose transport to skeletal muscle;^24,25^ further studies are needed to confirm whether sGC stimulators would have the same effect.

The sGC stimulator praliciguat was associated with skeletal muscle gene expression changes, and reduced fasting insulin, C-peptide, triglycerides, and insulin resistance, and increased energy expenditure in diet-induced obese mice.^26^ The precise mechanisms underlying the effects of sGC stimulators are not fully understood, but may involve both systemic factors, such as enhanced blood flow, as well as local effects. Studies in hepatic stellate cGKI-depleted mice have indicated that cGKI loss affects liver metabolism via a paracrine mechanism involving enhanced infiltration of macrophages and IL-6 signalling, and that this results in an impaired activity of insulin leading to fasting hyperglycaemia.^27^

Research in mice suggest that phosphodiesterase 9 (PDE9) is a regulator of energy metabolism,^28,29^ with evidence showing that both target disruption of the *PDE9* gene and PDE9 inhibition increase energy expenditure and mitochondrial activity in brown and white adipose tissue, thus conferring resistance to obesity induced by a high-fat diet.^28,29^

## Part 2. Pharmacological therapies that affect the NO–sGC–cGMP pathway but have not been indicated as a suitable therapy for HF

Despite approvals for erectile dysfunction (ED), pulmonary arterial hypertension (PAH) and benign prostatic hyperplasia, phosphodiesterase type 5 (PDE5) inhibitors have not been approved for the management of HF. Two meta-analyses examining the effect of PDE5 inhibitors on hemodynamic parameters showed an improvement in peak VO_2_ and LV ejection fraction (LVEF) in HFrEF but not HF with preserved ejection fraction (HFpEF), with no significant increase in adverse event rate.^30,31^ A reduction in hospitalisation rate was observed with PDE5 inhibitors across seven randomised controlled trials in HFrEF (relative risk, 0.340; 95% confidence interval, 0.140 to 0.820; P=0.02).^31^ A meta-analysis assessing the effects of PH-targeted therapies on exercise capacity in patients with HF, observed an improvement with PDE5 inhibitors in HFrEF.^32^ Additional evidence from large-scale randomised controlled trials is required to determine the efficacy of PDE5 inhibitors in the treatment of HFrEF. PDE5 inhibition is not associated with improved outcomes in HFpEF.^33-35^ The lack of benefit has been attributed to its inability to enhance cGMP production.^35^

Two multicentre studies with over 100 patients each have demonstrated a lack of improvement in exercise capacity orNT-proBNP levels with nitrates compared with placebo in patients with HFpEF.^36,37^

While sGC stimulators improve clinical outcomes in HFrEF, their efficacy has not been established in HFpEF. VITALITY-HFpEF demonstrated no improvement in the physical limitation score of the Kansas City Cardiomyopathy Questionnaire or 6-minute walk distance with vericiguat compared with placebo.^38^ In the phase II dose-finding SOCRATES-PRESERVED trial, 12 weeks’ vericiguat treatment did not reduce NT-proBNP levels or left atrial volume compared with placebo.^39^ The CAPACITY‑HFpEF study demonstrated that the sGC stimulator praliciguat had no significant effect on peak rate of oxygen consumption in patients with HFpEF.^40^

These findings suggest that reduced NO availability may not be a primary driver in the progression of HFpEF.^38,40^ The efficacy of sGC stimulators in isolated cells is diminished under the conditions of oxidative stress,^41^ which is often elevated in HFpEF due to metabolic comorbidities. This may help explain the limited effects of vericiguat and praliciguat on clinical outcomes observed in these studies.

Sacubitril-valsartan demonstrated improved clinical outcomes in patients with HF and New York Heart Association class II to III symptoms in the PARADIGM-HF trial^42^ but not in patients with advanced HFrEF or those following myocardial infarction (MI) with LV dysfunction without HF.^43^ In PARAGLIDE-HF, sacubitril-valsartan reduced plasma NT-proBNP levels in patients with worsening HF and ejection fraction >40% compared with valsartan alone.^44^ In the PARAGON trial, sacubitril-valsartan had no significant impact on the primary endpoint of total hospitalizations for HF and death from CV causes in patients with HF and ejection fraction ≥ 45%.^45^

**References**

1. Follmann M, Becker C, Rossig L, Sandner P, Stasch JP. Discovery and development of the soluble guanylate cyclase stimulator vericiguat for the treatment of chronic heart failure. In. *Contemporary Accounts in Drug Discovery and Development*: Wiley and Sons; 2021.

2. Stehle D, Xu MZ, Schomber T, Hahn MG, Schweda F, Feil S*, et al.* Novel soluble guanylyl cyclase activators increase glomerular cGMP, induce vasodilation and improve blood flow in the murine kidney. *Br J Pharmacol* 2022;**179**:2476-2489. doi:10.1111/bph.15586

3. Boden K, Sandner P, Roessig L, Vogel J, Chirinos JA, Mondritzki T. Vericiguat Improves Aortic Wave Reflection Parameters in a New Preclinical Model of Hypertension. *Circ Heart Fail* 2022;**15**:e008735. doi:10.1161/CIRCHEARTFAILURE.121.008735

4. Reinke Y, Gross S, Eckerle LG, Hertrich I, Busch M, Busch R*, et al.* The soluble guanylate cyclase stimulator riociguat and the soluble guanylate cyclase activator cinaciguat exert no direct effects on contractility and relaxation of cardiac myocytes from normal rats. *Eur J Pharmacol* 2015;**767**:1-9. doi:10.1016/j.ejphar.2015.09.022

5. Krishnan SM, Nordlohne J, Dietz L, Vakalopoulos A, Haning P, Hartmann E*, et al.* Assessing the Use of the sGC Stimulator BAY-747, as a Potential Treatment for Duchenne Muscular Dystrophy. *Int J Mol Sci* 2021;**22**:doi:10.3390/ijms22158016

6. Weber RE, Schulze KM, Colburn TD, Horn AG, Hageman KS, Ade CJ*, et al.* Capillary hemodynamics and contracting skeletal muscle oxygen pressures in male rats with heart failure: Impact of soluble guanylyl cyclase activator. *Nitric Oxide* 2022;**119**:1-8. doi:10.1016/j.niox.2021.12.001

7. Ahluwalia A, Foster P, Scotland RS, McLean PG, Mathur A, Perretti M*, et al.* Antiinflammatory activity of soluble guanylate cyclase: cGMP-dependent down-regulation of P-selectin expression and leukocyte recruitment. *Proceedings of the National Academy of Sciences of the United States of America* 2004;**101**:1386–1391. doi:10.1073/pnas.0304264101

8. Ferreira WA, Jr., Chweih H, Lanaro C, Almeida CB, Brito PL, Gotardo EMF*, et al.* Beneficial effects of soluble guanylyl cyclase stimulation and activation in sickle cell disease are amplified by hydroxyurea: In vitro and in vivo studies. *J Pharmacol Exp Ther* 2020;**374**:469–478. doi:10.1124/jpet.119.264606

9. Jungtanasomboon P, Nussaro S, Winwan H, Suebthawinkul P, Boonpala P, Dong VNK*, et al.* Vericiguat preserved cardiac function and mitochondrial quality in a rat model of mitral regurgitation. *Life Sci* 2023;**328**:121929. doi:10.1016/j.lfs.2023.121929

10. Defilippi CR, Alemayehu WG, Voors AA, Kaye D, Blaustein RO, Butler J*, et al.* Assessment of Biomarkers of Myocardial injury, Inflammation, and Renal Function in Heart Failure With Reduced Ejection Fraction: The VICTORIA Biomarker Substudy. *J Card Fail* 2023;**29**:448-458. doi:10.1016/j.cardfail.2022.12.013

11. Kramer F, Voss S, Roessig L, Igl BW, Butler J, Lam CSP*, et al.* Evaluation of high-sensitivity C-reactive protein and uric acid in vericiguat-treated patients with heart failure with reduced ejection fraction. *Eur J Heart Fail* 20201675–1683. doi:10.1002/ejhf.1787

12. Mauersberger C, Sager HB, Wobst J, Dang TA, Lambrecht L, Koplev S*, et al.* Loss of soluble guanylyl cyclase in platelets contributes to atherosclerotic plaque formation and vascular inflammation. *Nat Cardiovasc Res* 2022;**1**:1174–1186. doi:10.1038/s44161-022-00175-w

13. Hildebrand S, Ibrahim M, Schlitzer A, Maegdefessel L, Roll W, Pfeifer A. PDGF regulates guanylate cyclase expression and cGMP signaling in vascular smooth muscle. *Commun Biol* 2022;**5**:197. doi:10.1038/s42003-022-03140-2

14. Segura-Puimedon M, Mergia E, Al-Hasani J, Aherrahrou R, Stoelting S, Kremer F*, et al.* Proatherosclerotic Effect of the alpha1-Subunit of Soluble Guanylyl Cyclase by Promoting Smooth Muscle Phenotypic Switching. *Am J Pathol* 2016;**186**:2220-2231. doi:10.1016/j.ajpath.2016.04.010

15. Wobst J, von Ameln S, Wolf B, Wierer M, Dang TA, Sager HB*, et al.* Stimulators of the soluble guanylyl cyclase: promising functional insights from rare coding atherosclerosis-related GUCY1A3 variants. *Basic Res Cardiol* 2016;**111**:51. doi:10.1007/s00395-016-0570-5

16. Friebe A, Sandner P, Schmidtko A. cGMP: a unique 2nd messenger molecule – recent developments in cGMP research and development. *Naunyn-Schmiedeberg's Archives of Pharmacology* 2020;**393**:287–302. doi:10.1007/s00210-019-01779-z

17. Reiss C, Mindukshev I, Bischoff V, Subramanian H, Kehrer L, Friebe A*, et al.* The sGC stimulator riociguat inhibits platelet function in washed platelets but not in whole blood. *Br J Pharmacol* 2015;**172**:5199-5210. doi:10.1111/bph.13286

18. Hoffmann LS, Etzrodt J, Willkomm L, Sanyal A, Scheja L, Fischer AWC*, et al.* Stimulation of soluble guanylyl cyclase protects against obesity by recruiting brown adipose tissue. *Nat Commun* 2015;**6**:7235. doi:10.1038/ncomms8235

19. Hoffmann LS, Larson CJ, Pfeifer A. cGMP and Brown Adipose Tissue. *Handb Exp Pharmacol* 2016;**233**:283-299. doi:10.1007/164_2015_3

20. Sanyal A, Naumann J, Hoffmann LS, Chabowska-Kita A, Ehrlund A, Schlitzer A*, et al.* Interplay between Obesity-Induced Inflammation and cGMP Signaling in White Adipose Tissue. *Cell Rep* 2017;**18**:225-236. doi:10.1016/j.celrep.2016.12.028

21. Haas B, Mayer P, Jennissen K, Scholz D, Berriel Diaz M, Bloch W*, et al.* Protein kinase G controls brown fat cell differentiation and mitochondrial biogenesis. *Sci Signal* 2009;**2**:ra78. doi:10.1126/scisignal.2000511

22. Jennissen K, Siegel F, Liebig-Gonglach M, Hermann MR, Kipschull S, van Dooren S*, et al.* A VASP-Rac-soluble guanylyl cyclase pathway controls cGMP production in adipocytes. *Sci Signal* 2012;**5**:ra62. doi:10.1126/scisignal.2002867

23. Leiss V, Friebe A, Welling A, Hofmann F, Lukowski R. Cyclic GMP kinase I modulates glucagon release from pancreatic alpha-cells. *Diabetes* 2011;**60**:148-156. doi:10.2337/db10-0595

24. Etgen GJ, Jr., Fryburg DA, Gibbs EM. Nitric oxide stimulates skeletal muscle glucose transport through a calcium/contraction- and phosphatidylinositol-3-kinase-independent pathway. *Diabetes* 1997;**46**:1915-1919. doi:10.2337/diab.46.11.1915

25. Cidad P, Almeida A, Bolaños JP. Inhibition of mitochondrial respiration by nitric oxide rapidly stimulates cytoprotective GLUT3-mediated glucose uptake through 5'-AMP-activated protein kinase. *Biochem J* 2004;**384**:629-636. doi:10.1042/bj20040886

26. Schwartzkopf CD, Hadcock JR, Liu G, Germano P, Roux J, Shea CM*, et al.* Beneficial Metabolic Effects of Praliciguat, a Soluble Guanylate Cyclase Stimulator, in a Mouse Diet-Induced Obesity Model. *Front Pharmacol* 2022;**13**:852080. doi:10.3389/fphar.2022.852080

27. Lutz SZ, Hennige AM, Feil S, Peter A, Gerling A, Machann J*, et al.* Genetic ablation of cGMP-dependent protein kinase type I causes liver inflammation and fasting hyperglycemia. *Diabetes* 2011;**60**:1566-1576. doi:10.2337/db10-0760

28. Ceddia RP, Liu D, Shi F, Crowder MK, Mishra S, Kass DA*, et al.* Increased Energy Expenditure and Protection From Diet-Induced Obesity in Mice Lacking the cGMP-Specific Phosphodiesterase PDE9. *Diabetes* 2021;**70**:2823-2836. doi:10.2337/db21-0100

29. Mishra S, Sadagopan N, Dunkerly-Eyring B, Rodriguez S, Sarver DC, Ceddia RP*, et al.* Inhibition of phosphodiesterase type 9 reduces obesity and cardiometabolic syndrome in mice. *J Clin Invest* 2021;**131**:doi:10.1172/JCI148798

30. Zhuang XD, Long M, Li F, Hu X, Liao XX, Du ZM. PDE5 inhibitor sildenafil in the treatment of heart failure: a meta-analysis of randomized controlled trials. *International journal of cardiology* 2014;**172**:581-587. doi:10.1016/j.ijcard.2014.01.102

31. Hwang IC, Kim YJ, Park JB, Yoon YE, Lee SP, Kim HK*, et al.* Pulmonary hemodynamics and effects of phosphodiesterase type 5 inhibition in heart failure: a meta-analysis of randomized trials. *BMC Cardiovasc Disord* 2017;**17**:150. doi:10.1186/s12872-017-0576-4

32. Guay CA, Morin-Thibault LV, Bonnet S, Lacasse Y, Lambert C, Lega JC*, et al.* Pulmonary hypertension-targeted therapies in heart failure: A systematic review and meta-analysis. *PLoS One* 2018;**13**:e0204610. doi:10.1371/journal.pone.0204610

33. Hussain I, Mohammed SF, Forfia PR, Lewis GD, Borlaug BA, Gallup DS*, et al.* Impaired right ventricular-pulmonary arterial coupling and effect of sildenafil in heart failure with preserved ejection fraction: an ancillary analysis from the phosphodiesterase-5 inhibition to improve clinical status and exercise capacity in diastolic heart failure (RELAX) trial. *Circ Heart Fail* 2016;**9**:e002729. doi:10.1161/circheartfailure.115.002729

34. Liu LC, Hummel YM, van der Meer P, Berger RM, Damman K, van Veldhuisen DJ*, et al.* Effects of sildenafil on cardiac structure and function, cardiopulmonary exercise testing and health-related quality of life measures in heart failure patients with preserved ejection fraction and pulmonary hypertension. *Eur J Heart Fail* 2017;**19**:116–125. doi:10.1002/ejhf.662

35. Redfield MM, Chen HH, Borlaug BA, Semigran MJ, Lee KL, Lewis G*, et al.* Effect of phosphodiesterase-5 inhibition on exercise capacity and clinical status in heart failure with preserved ejection fraction: a randomized clinical trial. *Jama* 2013;**309**:1268–1277. doi:10.1001/jama.2013.2024

36. Borlaug BA, Anstrom KJ, Lewis GD, Shah SJ, Levine JA, Koepp GA*, et al.* Effect of inorganic nitrite vs placebo on exercise capacity among Patients with heart failure with preserved ejection fraction: the INDIE-HFpEF randomized clinical trial. *Jama* 2018;**320**:1764–1773. doi:10.1001/jama.2018.14852

37. Redfield MM, Anstrom KJ, Levine JA, Koepp GA, Borlaug BA, Chen HH*, et al.* Isosorbide mononitrate in heart failure with preserved ejection fraction. *N Engl J Med* 2015;**373**:2314–2324. doi:10.1056/NEJMoa1510774

38. Armstrong PW, Lam C, Anstrom K, Ezekowitz J, Hernandez A, O’Connor C*, et al.* Effect of vericiguat vs placebo on quality of life in patients with heart failure and preserved ejection fraction: The VITALITY-HFpEF randomized clinical trial. *JAMA* 2020;**324**:1512–1521. doi:10.1001/jama.2020.15922

39. Pieske B, Maggioni AP, Lam CSP, Pieske-Kraigher E, Filippatos G, Butler J*, et al.* Vericiguat in patients with worsening chronic heart failure and preserved ejection fraction: results of the SOluble guanylate Cyclase stimulatoR in heArT failurE patientS with PRESERVED EF (SOCRATES-PRESERVED) study. *Eur Heart J* 2017;**38**:1119–1127. doi:10.1093/eurheartj/ehw593

40. Udelson JE, Lewis GD, Shah SJ, Zile MR, Redfield MM, Burnett J, Jr.*, et al.* Effect of praliciguat on peak rate of oxygen consumption in patients with heart failure with preserved ejection fraction: the CAPACITY HFpEF randomized clinical trial. *JAMA* 2020;**324**:1522–1531. doi:10.1001/jama.2020.16641

41. Sandner P, Follmann M, Becker-Pelster E, Hahn MG, Meier C, Freitas C*, et al.* Soluble GC stimulators and activators: past, present and future. *Br J Pharmacol* 2021;**doi: 10.1111/bph.15698**:doi:10.1111/bph.15698

42. McMurray JJ, Packer M, Desai AS, Gong J, Lefkowitz MP, Rizkala AR*, et al.* Angiotensin-neprilysin inhibition versus enalapril in heart failure. *N Engl J Med* 2014;**371**:993–1004. doi:10.1056/NEJMoa1409077

43. Bozkurt B, Nair AP, Misra A, Scott CZ, Mahar JH, Fedson S. Neprilysin inhibitors in heart failure: the science, mechanism of action, clinical studies, and unanswered questions. *JACC Basic Transl Sci* 2023;**8**:88–105. doi:10.1016/j.jacbts.2022.05.010

44. Mentz RJ, Ward JH, Hernandez AF, Lepage S, Morrow DA, Sarwat S*, et al.* Angiotensin-Neprilysin Inhibition in Patients With Mildly Reduced or Preserved Ejection Fraction and Worsening Heart Failure. *J Am Coll Cardiol* 2023;**82**:1-12. doi:10.1016/j.jacc.2023.04.019

45. Solomon SD, McMurray JJV, Anand IS, Ge J, Lam CSP, Maggioni AP*, et al.* Angiotensin-neprilysin inhibition in heart failure with preserved ejection fraction. *N Engl J Med* 2019;**381**:1609–1620. doi:10.1056/NEJMoa1908655
